# Relationship of the Esophageal Microbiome and Tissue Gene Expression and Links to the Oral Microbiome: A Randomized Clinical Trial

**DOI:** 10.14309/ctg.0000000000000235

**Published:** 2020-12-07

**Authors:** Medini K. Annavajhala, Michael May, Griselda Compres, Daniel E. Freedberg, Roseanna Graham, Stephania Stump, Jianwen Que, Tal Korem, Anne-Catrin Uhlemann, Julian A. Abrams

**Affiliations:** 1 Department of Medicine, Columbia University Irving Medical Center, New York, New York, USA;; 2 Microbiome and Pathogen Genomics Core, Department of Medicine, Columbia University Irving Medical Center, New York, New York, USA;; 3 College of Dental Medicine, Columbia University, New York, New York, USA;; 4 Department of Systems Biology, Columbia University, New York, New York, USA.

## Abstract

**INTRODUCTION:**

Although the microbiome is altered in various esophageal diseases, there is no direct evidence for a link between the oral or esophageal microbiome and underlying esophageal tissue. Here, we aimed to address these gaps through use of an antimicrobial mouth rinse to modify the esophageal microbiome and tissue gene expression.

**METHODS:**

In this randomized controlled trial, patients scheduled to undergo endoscopy for clinical indications used chlorhexidine mouth rinse or no treatment for 2 weeks before endoscopy. Oral swabs and saliva were collected at baseline and at follow-up, and the esophagus was sampled on the day of endoscopy. The microbiome was analyzed by 16S rRNA gene sequencing, and esophageal tissue gene expression was ascertained by RNA-Seq.

**RESULTS:**

Twenty subjects were enrolled and included in the analyses. Within individuals, the oral and esophageal microbiome composition was significantly correlated. Chlorhexidine treatment associated with significant alterations to the relative abundance of several esophageal bacterial taxa, and to expression of genes in the esophagus including reductions in periostin, claudin-18, chemokines *CXCL1* and *CXCL13*, and several members of the tumor necrosis factor receptor superfamily. A taxon in genus *Haemophilus* in the esophagus also associated with significant changes in tissue gene expression.

**DISCUSSION:**

The oral and esophageal microbiomes are closely related within individuals, and esophageal microbiome alterations correlate with tissue gene expression changes. The esophageal microbiome may act as an important cofactor that influences pathogenesis and outcomes of diseases such as eosinophilic esophagitis, gastroesophageal reflux, and Barrett's esophagus.

## INTRODUCTION

The overall composition of the esophageal microbiome resembles the oral microbiome, with a predominance of Firmicutes and the genus *Streptococcus* in particular (1,2). There is an emerging body of literature to suggest that the esophageal microbiome is altered in various esophageal disease states, such as Barrett's esophagus (BE) and eosinophilic esophagitis (EoE) (3–8). Oral microbiome alterations have been described to precede the development of esophageal adenocarcinoma (9), and our group has previously reported that there are marked oral microbiome alterations in patients with BE (10). However, although similarities between oral and esophageal microbial communities have been described separately in previous studies, a direct link between the communities within individuals has yet to be established. Furthermore, there is no clear evidence that elements of the esophageal microbiome interact with esophageal tissue, or whether they simply represent transient colonizers. This dearth of knowledge is due in part to challenges to studying the oral and upper gastrointestinal microbiome in animal models, as well as the need for an invasive procedure (i.e., upper endoscopy) to directly sample the esophagus.

The gut microbiome in the colon closely interacts with the host epithelium, microenvironment, and immune system (11,12). If the esophageal microbiome is similarly a cofactor for maintenance of esophageal health, and alterations to the microbiome predispose to esophageal disease, then this microbiome would represent a new therapeutic target. Developing therapies that alter the esophageal microbiome directly is challenging. If there is a direct link between the mouth and esophagus, then targeting the oral microbiome may represent an easily accessible means of altering the esophageal microbiome.

We hypothesized that changes to the oral microbiome impact the esophageal microbiome, and that these alterations can in turn influence esophageal gene expression. To test this hypothesis, we conducted a proof-of-concept randomized controlled trial of an antimicrobial mouth rinse in patients scheduled to undergo upper endoscopy and assessed the effects on the oral and esophageal microbiome and esophageal tissue gene expression.

## METHODS

### Study design

This was an unblinded, randomized controlled trial of adults 18 years and older scheduled to undergo upper endoscopy for clinical indications (see Supplementary Table 1, Supplementary Digital Content 3, http://links.lww.com/CTG/A461). Subjects were excluded for any of the following reasons: use of proton pump inhibitors (PPIs) or H2-receptor antagonists beginning ≤1 month before enrollment (standing acid suppressant use >1 month before enrollment was permitted); history of upper gastrointestinal cancer or histologically proven BE; and history of antireflux, bariatric, or other gastric or esophageal surgery. Details of additional study criteria, data collected, and ethical considerations are provided in Supplemental Methods (see Supplementary Digital Content 1, http://links.lww.com/CTG/A459).

### Randomization and intervention

Chlorhexidine was chosen for this study based on its broad bactericidal effects and established excellent safety profile. Subjects were randomized 1:1 to receive chlorhexidine mouth rinse or no treatment. Randomization was performed in blocks of 4, with randomization stratified by PPI use. Those assigned to the treatment arm used chlorhexidine 0.12% mouth rinse twice daily for the 2 weeks before the endoscopy. Subjects were instructed to rinse in the morning and evening after brushing teeth, with 15-mL chlorhexidine for 30 seconds and then spitting out the rinse. Subjects were specifically instructed not to swallow the mouth rinse. Patients in the no treatment arm were instructed not to use any mouth rinse during the study period. Adverse events and tooth discoloration were assessed at baseline and at follow-up (see Supplemental Methods, Supplementary Digital Content 1, http://links.lww.com/CTG/A459).

### Biosample collection and microbiome and gene expression analyses

Full details of biosample collection and microbiome analyses are provided in Supplemental Methods (see Supplementary Digital Content 1, http://links.lww.com/CTG/A459). All subjects were fasting (nothing to eat or drink after midnight the night before) at the time of sample collections. Saliva and oral swabs were collected at baseline (2 weeks before the endoscopy) and on the day of endoscopy. During the endoscopy, esophageal brushings were collected from the distal esophagus. 16S rRNA gene sequencing was performed of the V3/V4 hypervariable region. Standard α-diversity and β-diversity as well as differential abundance analyses were performed. Analyses were performed to determine how closely related the oral and esophageal microbial communities were within individuals. Analyses were also performed to determine whether the relative abundances of specific taxa in the esophagus were associated with the relative abundances for the same taxa in oral swabs and in saliva. Correlation of individual operational taxonomic units (OTUs) between sampling sites (esophagus-oral swab and esophagus-saliva) within individuals was also assessed. Esophageal microbiome composition was compared after intervention based on treatment arm.

Esophageal tissue gene expression analyses were performed using RNA-Seq. Full details are provided in Supplemental Methods (see Supplementary Digital Content 1, http://links.lww.com/CTG/A459). Differential gene expression was compared between treatment arms. To link the presence and relative abundance of specific microbiota with tissue gene expression data, Mantel tests were run on full OTU count tables and count tables for differentially expressed genes as identified above, across treatment groups at each site.

### Other statistical analyses

Proportions and mean values or medians were calculated to summarize the data. Categorical variables were compared between groups using Fisher exact tests, and continuous variables were compared using rank sum or *t* tests when appropriate. Statistical significance was defined as *P* < 0.05. There were little data on which to base sample size calculations for the design of this study in terms of the effect size of chlorhexidine on the microbiome assessed by 16S rRNA gene sequencing and on esophageal tissue gene expression assessed by RNA-Seq. Analyses were performed using Stata 14.0 and R.

## RESULTS

A total of 20 patients were enrolled, and all completed the study (see Supplementary Figure 1, Supplementary Digital Content 2, http://links.lww.com/CTG/A460). Ten patients were assigned to the chlorhexidine arm, and 10 patients were assigned to the no treatment arm. The patient characteristics are shown in Supplementary Table 1 (see Supplementary Digital Content 3, http://links.lww.com/CTG/A461). There were no significant differences between the 2 arms in terms of age, sex, ethnicity/race, PPI use, aspirin use, dietary fat or fiber intake, or history of gastroesophageal reflux disease. There were no reported side effects associated with chlorhexidine use, and no subjects in the chlorhexidine arm had a worsening of tooth discoloration.

At baseline, there were no notable differences between the chlorhexidine and no treatment arms in the oral microbiome from saliva or oral swabs, demonstrating that randomization was effective at eliminating major microbiome differences between treatment arms. Comparing the 2 arms, there was no difference in baseline richness or diversity in saliva (Chao, *P* = 0.71; Shannon index, *P* = 1.00) or in oral swabs (Chao, *P* = 0.88; Shannon index, *P* = 0.82). Similarly, no differentially abundant taxa were identified in saliva or in oral swabs between the 2 arms at baseline (see Supplementary Table 2, Supplementary Digital Content 4, http://links.lww.com/CTG/A462).

### Similarity of the oral and esophageal microbiome within individuals

**Figure 1.**
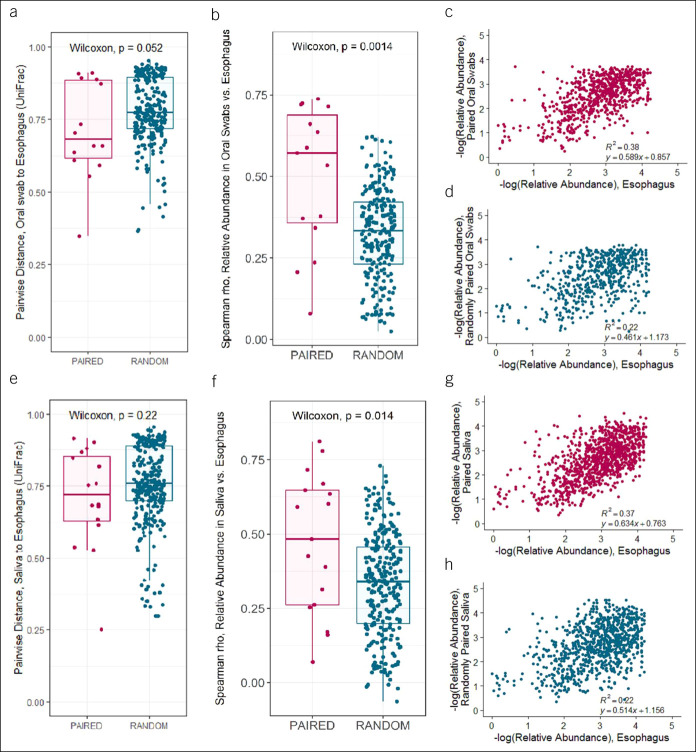
The microbiome from oral swabs and esophageal brushings were closely related within individuals, with a modestly weaker association between saliva and esophagus microbiome. *Oral swab-esophagus*: (**a**) UniFrac distances between oral swabs and esophageal brushings were smaller in paired samples within individuals compared with randomly matched samples from different individuals (rank sum *P* = 0.052); (**b**) within-sample pair correlations of relative abundance of top 100 OTUs, showing correlation coefficients for each sample pair, demonstrate significantly higher correlations within individuals compared with sample pairs from randomly matched individuals (rank sum *P* = 0.0014); dot-plot showing that correlations of individual OTU relative abundance in (**c**) paired samples within individuals (*R*^2^ = 0.38) are numerically higher than in (**d**) paired samples from randomly paired individuals (*R*^2^ = 0.22). *Saliva-esophagus*: Comparisons of paired samples within individuals with paired samples from randomly matched individuals showed (**e**) no significant difference in UniFrac distances; (**f**) significantly higher correlations within individuals at the OTU level (rank sum *P* = 0.014); numerically correlations of individual OTUs in (**g**) paired samples (*R*^2^ = 0.37) compared with (**h**) randomly paired samples (*R*^2^ = 0.22). OTU, operational taxonomic unit.

To assess the relationship between the oral and esophageal microbiome, within-individual correlations were assessed at the end of the study period between contemporaneously collected oral swabs and esophageal brushings and between saliva and esophageal brushings. On β-diversity analyses, paired microbiome samples of oral swabs and esophagus were more closely related within individuals compared with randomly paired samples (*P* = 0.052; Figure 1a). Extending these analyses to the taxonomic level, there was significant correlation between the relative abundance of taxa in paired oral swab and esophageal samples within individuals (Figure 1b–d). Overall, similar results were seen comparing paired microbiome samples of saliva and esophagus with randomly paired samples, except there was no significant difference in beta diversity analyses (Figure 1e–h). Within individuals, taxa that were significantly correlated between sites included OTU5 (genus *Fusobacterium*; oral swab-esophagus *R*^2^ = 0.77, adj. *P* < 0.01; saliva-esophagus *R*^2^ = 0.72, adj. *P* < 0.01), OTU8 (family *Carnobacteriaceae*; saliva-esophagus *R*^2^ = 0.46, adj. *P* = 0.02), OTU11 (genus *Porphyromonas*; oral swab-esophagus *R*^2^ = 0.43, adj. *P* = 0.04), OTU14 (family *Pasteurellaceae*; saliva-esophagus *R*^2^ = 0.82, adj. *P* < 0.01), OTU16 (order *Bacteroidales*; oral swab-esophagus *R*^2^ = 0.59, adj. *P* < 0.01), and OTU21 (genus *Prevotella*; saliva-esophagus *R*^2^ = 0.68, adj. *P* < 0.01) (see Supplementary Table 3, Supplementary Digital Content 5, http://links.lww.com/CTG/A463, for full list).

### Effects of chlorhexidine on the oral microbiome

**Figure 2.**
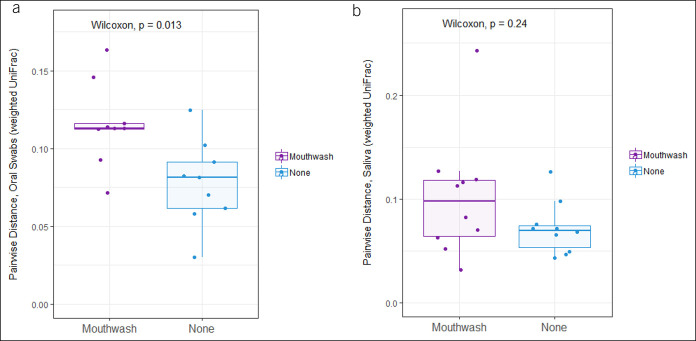
Treatment with chlorhexidine caused significant alterations to the oral microbiome assessed by oral swabs, but not saliva, based on weighted β-diversity analyses. Comparisons shown are of paired pre-treatment and post-treatment samples in patients in the chlorhexidine and no-treatment arms for (**a**) oral swabs (rank sum *P* = 0.013) and (**b**) saliva (rank sum *P* = 0.24).

Over the 2-week study period, in both the chlorhexidine and no treatment arms, there were no significant differences in within-individual changes in α-diversity measures in either oral swabs or saliva (see Supplementary Figure 2A–F, Supplementary Digital Content 2, http://links.lww.com/CTG/A460). However, chlorhexidine treatment effect was seen in weighted β-diversity analyses of oral swabs but not saliva; comparing the microbiome at end-of-study to beginning-of-study, there was a significantly greater change to the oral microbiome within individuals in the chlorhexidine arm compared with the no treatment arm (Figure 2). In the no treatment arm, there were no significant alterations to the relative abundance of taxa over the 2-week period in oral swabs or saliva. In the chlorhexidine arm comparing after vs before treatment, relatively minimal changes were observed in specific taxa after adjustments for multiple comparisons; OTU18 (genus *Rothia*) was reduced in saliva, and there were no significantly altered OTUs in oral swabs. Comparing the arms after the 2-week period, OTU380 (genus *Capnocytophaga*) was significantly increased in oral swabs in the chlorhexidine arm, with no significant alterations seen in saliva (see Supplementary Table 4, Supplementary Digital Content 6, http://links.lww.com/CTG/A464).

### Effects of chlorhexidine on the esophageal microbiome

Based on the high within-individual correlation between the oral and esophageal microbiome, together with effective randomization as demonstrated by similar baseline oral microbiome between treatment arms, we assumed that there were no major differences in the esophageal microbiome at baseline between the 2 treatment arms. Thus, end-of-study comparisons of the esophageal microbiome between the 2 arms would be representative of treatment effect of chlorhexidine.

**Figure 3.**
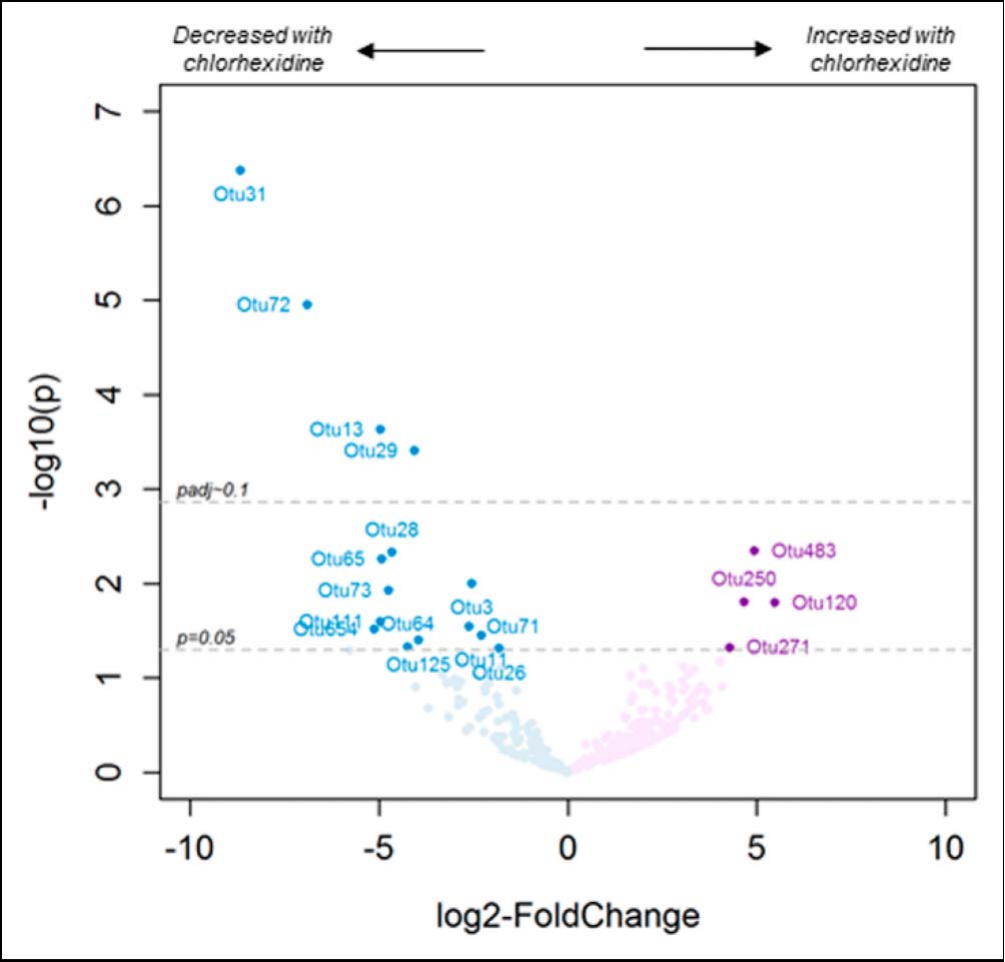
Volcano plots showing differentially abundant OTUs in the esophagus comparing the 2 study arms at the end of the treatment period. For each plot, dots on the left represent taxa decreased in the chlorhexidine arm, and dots on the right represent taxa increased in the chlorhexidine arm. See Supplementary Table 4 (Supplementary Digital Content 6, http://links.lww.com/CTG/A464) for list of specific OTUs altered in the esophagus, oral swabs, and saliva. OTU, operational taxonomic unit.

Comparing the chlorhexidine with no treatment arms, there were no differences in esophageal microbiome α-diversity (Chao, *P* = 0.77; Shannon index, *P* = 0.34) (see Supplementary Figure 2G–I, Supplementary Digital Content 2, http://links.lww.com/CTG/A460). After the 2-week treatment period, there was some clustering based on treatment arm which did not achieve significance at *P* < 0.05 (unweighted, permutational multivariate ANOVA [PERMANOVA] *P* = 0.08; weighted, PERMANOVA *P* = 0.07) (see Supplementary Figure 3, Supplementary Digital Content 2, http://links.lww.com/CTG/A460). Chlorhexidine treatment resulted in significantly reduced relative abundance of 3 taxa: OTU 29 (genus *Haemophilus*); OTU 72 (genus *Veillonella*); and OTU 31 (phylum SR1) (Figure 3 and see Supplementary Table 4, Supplementary Digital Content 6, http://links.lww.com/CTG/A464).

### Changes to esophageal tissue gene expression

**Figure 4.**
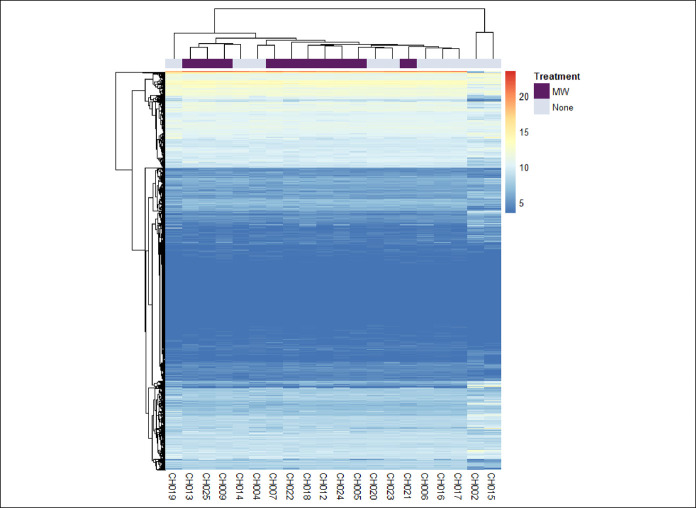
Hierarchical clustering of gene expression based on the top 100 differentially expressed genes based on RNA-Seq analyses. Subjects labeled with purple are in the chlorhexidine (MW) arm, and those labeled light blue are in the no treatment (no MW) arm.

Analyses were then performed using RNA-Seq of esophageal squamous biopsies that were collected at the same time as the esophageal brushings, to determine whether treatment with chlorhexidine mouth rinse was associated with changes in esophageal gene expression. Hierarchical unsupervised clustering analyses were suggestive of clustering by treatment arm (Figure 4). A total of 270 genes had significantly altered expression (adjusted *P* < 0.10), 67 of which had an adjusted *P* value <0.05 (see Supplementary Table 5, Supplementary Digital Content 7, http://links.lww.com/CTG/A465). Notable genes included marked reductions in expression of periostin (log-fold change −4.75, adj *P* = 0.0003), claudin-18 (log-fold change −8.01, adj *P* = 0.002), histidine decarboxylase (log-fold change −4.46, adj *P* = 0.0004), and chemokines *CXCL1* (log-fold change −3.34, adj *P* = 0.048) and *CXCL13* (log-fold change −6.05, adj *P* = 0.015). There were also significant reductions in the expression of several members of the tumor necrosis factor receptor superfamily, including *TNFRSF4*, *TNFRSF8*, *TNFRSF14*, *TNFRSF17*, and *TNFRSF18*.

### Relationship between the esophageal microbiome and tissue gene expression

**Figure 5.**
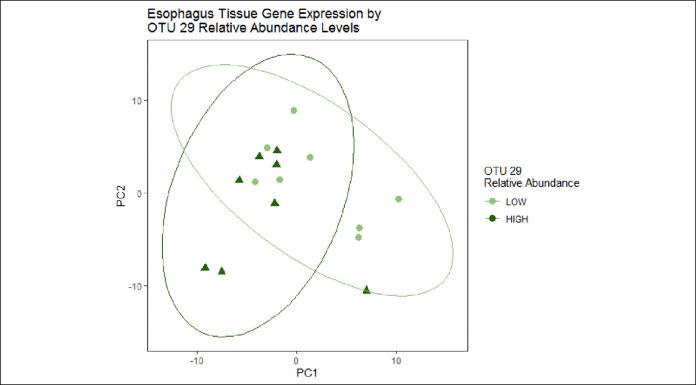
Principal coordinate analysis of esophageal tissue gene expression demonstrates significant clustering by relative abundance of OTU29 (genus *Haemophilus*) in the esophagus (permutational multivariate ANOVA *P* = 0.017). High = above the median; low = below the median.

Analyses were then performed to determine whether specific oral or esophageal microbiome characteristics were associated with esophageal tissue gene expression changes. Of the 3 sampling sites, the microbiome of the esophagus correlated most strongly with esophageal tissue gene expression (weighted UniFrac: Mantel *R*^2^ = 0.25, Mantel *P* = 0.12; Bray-Curtis: Mantel *R*^2^ = 0.27, Mantel *P* = 0.08). Exploratory analyses were performed to assess associations between tissue gene expression and relative abundance of OTU29 (genus *Haemophilus*), as this taxon was significantly altered in the esophagus by chlorhexidine treatment, and *Haemophilus* is present in the mouth and is increased in active EoE (8,13). When grouped by OTU29 relative abundance (above or below the median), there was significant clustering in principal coordinates analyses of tissue gene expression (PERMANOVA *P* = 0.017) (Figure 5). A total of 373 genes were differentially expressed comparing high vs low OTU29 relative abundance but not by treatment arm (see Supplementary Table 6, Supplementary Digital Content 8, http://links.lww.com/CTG/A466). Notable examples of these genes included increased expression of cytokines *CCL3*, *CCL18*, and *CXCL12*, B cell markers *CD19* and *CD22*, and autophagy regulators death-associated protein kinase 1 and 2 (*DAPK1* and *DAPK2*) and cysteine protease ATG4B (*ATG4B*), as well as markedly decreased keratin-1 (*KRT1*) expression.

## DISCUSSION

The results of this randomized controlled trial demonstrate that there is a direct link between the oral and esophageal microbiome. Within individuals, the overall bacterial composition of these 2 sites was highly similar, and treatment with an antibacterial mouth rinse altered the microbial communities of both sites. Chlorhexidine-induced alterations to the esophageal microbiome also correlated with marked changes in esophageal tissue gene expression. Although the clinical implications of these findings remain to be determined, the results of this proof-of-concept study lend credence to the idea that the esophageal microbiome is a potential mediator of esophageal health and disease. Furthermore, treatments that change the oral microbiome could possibly influence esophageal microbial composition and underlying biology.

It is interesting to note that the oral microbiome assessed by oral swabs compared with saliva was more closely linked to esophageal bacterial composition. Oral swabs presumably sample bacteria that reside in biofilms and that interact with underlying tissue, and bacteria with similar properties may also populate the esophagus via biofilms. Oral biofilms can harbor pathobionts and promote gingivitis and other periodontal diseases. In *in vitro* experiments using organotypic gingiva, exposure to oral flora resulted in increased epithelial thickness, keratinocyte proliferation, and inflammatory cytokine production (14). In the colon, biofilms are present on proximal tumors and adjacent paired normal mucosa (15) and promote the development of colonic neoplasia in mice (16). Our group recently described microbiome alterations that occur with progression from BE to esophageal adenocarcinoma, including increases in *Enterobacteriaceae* (17), and biofilms harboring these bacteria may promote neoplasia.

Chlorhexidine mouth rinse exerted a significant impact on the oral swab microbiome, but had a limited effect on the salivary microbiome. This finding is consistent with previous studies; Yamanaka et al. demonstrated that periodontal therapy such as tooth cleaning and plaque removal significantly altered the supragingival microbiome with only minimal effects on the salivary microbiome. The salivary microbiome may serve as a good biomarker for conditions such as BE (10), as its composition is stable over time and relatively unperturbed in the face of exposures (18,19).

Concurrent with shifts in the esophageal microbiome, chlorhexidine treatment changed expression in several genes associated with inflammation, including reduced expression of multiple members of the tumor necrosis factor receptor superfamily as well as several chemokines integral to the host inflammatory response to pathogens (20). Periostin and claudin-18 expression were also significantly reduced; periostin stimulates eosinophil recruitment and adhesion, promotes subepithelial fibrosis in EoE, and decreases in EoE after treatment with topical steroids (21–25). Claudins are key components to epithelial tight junctions, and claudin-18 has been reported to be highly expressed in BE and may be associated with increased resistance to acid reflux (26,27). In sum, these data suggest that bacteria may be important mediators of esophageal inflammation, and microbiome modification represents a novel approach to decrease inflammation and potentially modify esophageal disease risk.

A notable esophageal microbiome alteration was a reduction in the relative abundance of an OTU assigned to genus *Haemophilus*. *Haemophilus parainfluenzae* and *H. haemolyticus* are native to the oral cavity (13). Although not commonly associated with enteric infections, certain *Haemophilus* species can adhere to intestinal mucosa (28). Harris et al. (8) reported increased relative abundance of *Haemophilus* in the esophagus of patients with untreated EoE, with decreases observed after treatment with topical steroids or dietary modification. We observed broad changes in esophageal gene expression associated with relative abundance of this taxon, a majority of which seemed to be independent of the effects of chlorhexidine. High relative abundance was associated with increased expression of *OSGIN1*, a transcriptional target of *NRF2* (29). The *NRF2* pathway plays an important role in basal cell hyperplasia in EoE (30), raising the possibility that *Haemophilus* species could play a biological role in EoE.

There are a several mechanisms by which treatment with antimicrobial mouth rinse could impact esophageal tissue gene expression. Bacteria may interact directly with underlying epithelium; increased toll-like receptor expression has been observed in patients with active EoE (31), and LPS exposure to BE cells induces expression of proinflammatory cytokines in BE cells *in vitro* (32). Microbiome alterations may result in changes to local metabolite production. There were nonsignificant increases in oral microbial tryptophan metabolism in the chlorhexidine arm, and tryptophan metabolites such as indole-3-aldehyde have anti-inflammatory effects through induction of interleukin-22 and other mechanisms (33). Although it is possible that chlorhexidine induced all the esophageal tissue gene expression alterations independent of effects on the microbiome, we believe that this is unlikely.

There were several strengths to this study. This was a randomized trial in humans of an intervention aimed at altering the microbiome. The randomization was effective at the microbiome level, minimizing between-group heterogeneity that plagues observational studies and allowing between-arm comparisons in the current study. The oral and esophageal microbiome was assessed in individuals simultaneously, demonstrating a close link in the bacterial make-up of the 2 sites. Finally, using RNA-Seq, we were able to gain initial insights into the nature of the relationship between the microbiome and esophagus tissue gene expression, despite the low bacterial density of this body site.

There were also certain limitations. Repeated endoscopies were not clinically indicated, and thus, esophageal analyses were restricted to assessment at a single time point. As such, we were not able to assess within-individual changes. However, at baseline, there were no differences in the oral microbiome between the 2 arms, demonstrating that randomization eliminated appreciable microbiome differences between the groups. Furthermore, there was a close link between the microbiome composition of the mouth and esophagus within individuals. As such, between-arm comparisons of the esophagus most likely reflected the effects of chlorhexidine treatment. It is possible that, although subjects were instructed to gargle and spit the chlorhexidine, some amount of swallowed mouth rinse may have directly altered the esophageal microbiome. Regardless of the exact mechanisms, alterations in esophageal microbial composition correlated with changes to esophageal tissue gene expression. Finally, the study had a relatively small sample size, limiting the power to detect more subtle effects of chlorhexidine on the microbiome.

In conclusion, this proof-of-concept randomized trial of treatment with an antimicrobial mouth rinse demonstrated that the oral and esophageal microbiome are related, and that alterations to the esophageal microbiome correlate with changes to esophageal tissue gene expression. These findings implicate the esophageal microbiome as a potentially important cofactor in esophageal homeostasis. In addition, the oral and esophageal microbiome may represent novel therapeutic targets to influence risk of and outcomes of diseases of the esophagus such as EoE, gastroesophageal reflux, and BE.

## CONFLICTS OF INTEREST

**Guarantor of the article:** Julian A. Abrams, MD, MS.

**Specific author contributions:** M.K.A.: data curation, formal analysis, methodology, software, validation, manuscript drafting, and preparation. M.M., G.C., and D.E.F.: investigation, manuscript review, and editing. R.G.: manuscript review and editing. T.K.: formal analysis, methodology, supervision, manuscript review, and editing. S.S.: investigation, manuscript review, and editing. A.-C.U.: methodology, resources, supervision, manuscript review, and editing. J.A.A.: conceptualization of study, formal analysis, funding acquisition, investigation, methodology, project administration, resources, supervision, manuscript drafting, and preparation.

**Financial support:** Funded in part by NIH K23 DK111847 (D.E.F.), NIH R01 AI116939 (A.-C.U.), NIH U54 CA163004 (J.A.A.), and NIH R01 CA238433 (J.A.A.).

**Potential competing interests:** None to report.

**Trial registration:** The study was registered with ClinicalTrials.gov (NCT02513784).

**Study Highlights**

**WHAT IS KNOWN**

✓ The overall composition of the esophageal microbiome resembles the oral microbiome.
✓ Esophageal microbiome alterations have been associated with a variety of esophageal diseases.

**WHAT IS NEW HERE**

✓ Within individuals, the oral and esophageal microbiome composition is closely related.
✓ Alterations to the esophageal microbiome correlate with pronounced changes to esophageal tissue gene expression.

**TRANSLATIONAL IMPACT**

✓ Treatments aimed at altering the oral and esophageal microbiome may represent a novel therapeutic approach to influence risk of and outcomes of diseases of the esophagus.

## Supplementary Material

SUPPLEMENTARY MATERIAL
