# Supplementary material for: Relationship of the Esophageal Microbiome and Tissue Gene Expression and Links to the Oral Microbiome: A Randomized Clinical Trial
Source: Clin Transl Gastroenterol. 2020 Dec 7;11(12):e00235. doi: 10.14309/ctg.0000000000000235 (PMC7721221; doi:10.14309/ctg.0000000000000235)
Supplement: SUPPLEMENTARY MATERIAL [file ct9-11-e00235-s001.pdf]

SUPPLEMENTARY FIGURES

Supplementary Figure 1.

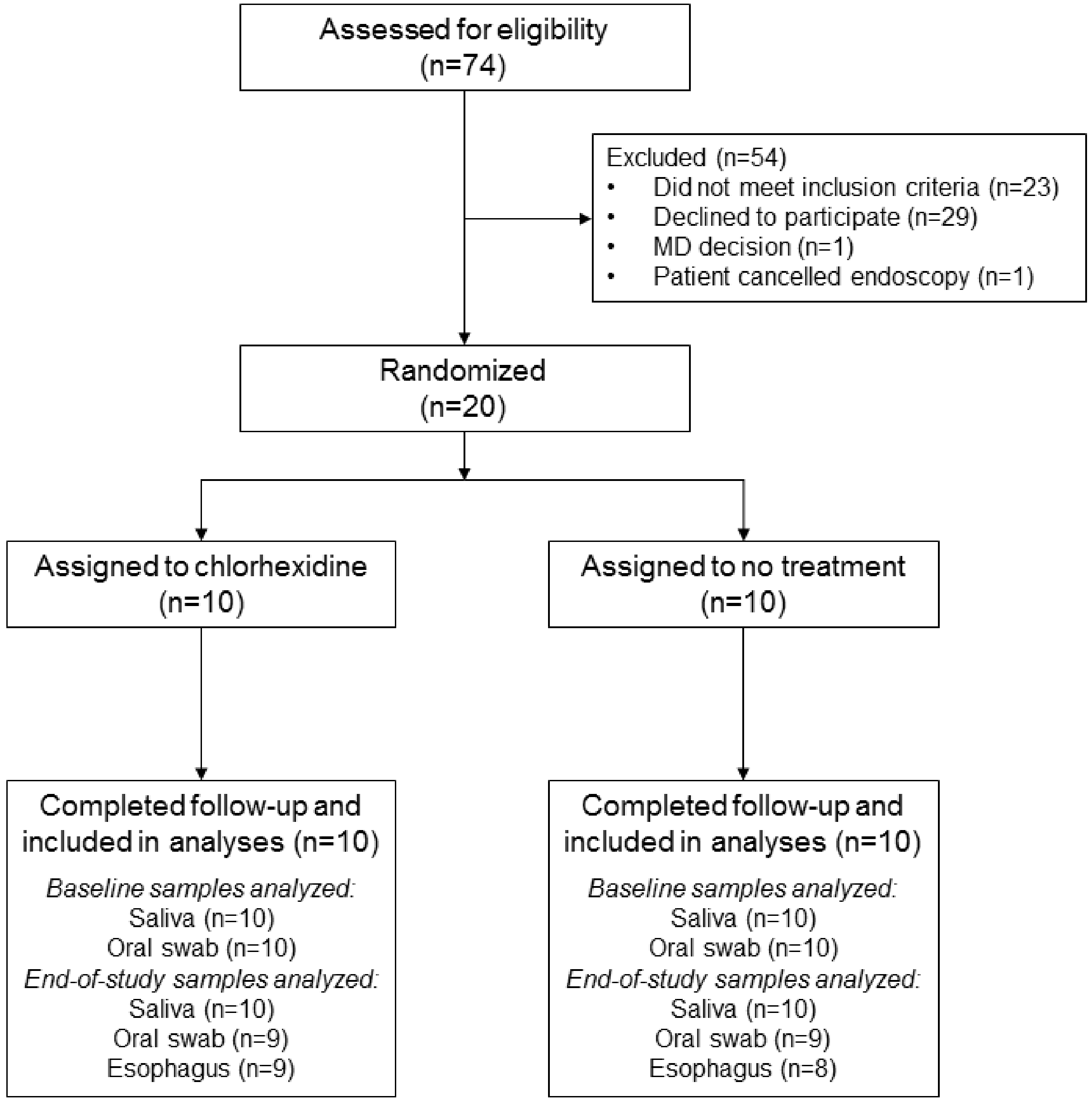

Supplementary Figure 1. Flow diagram of patients in the trial.

Supplementary Figure 2.

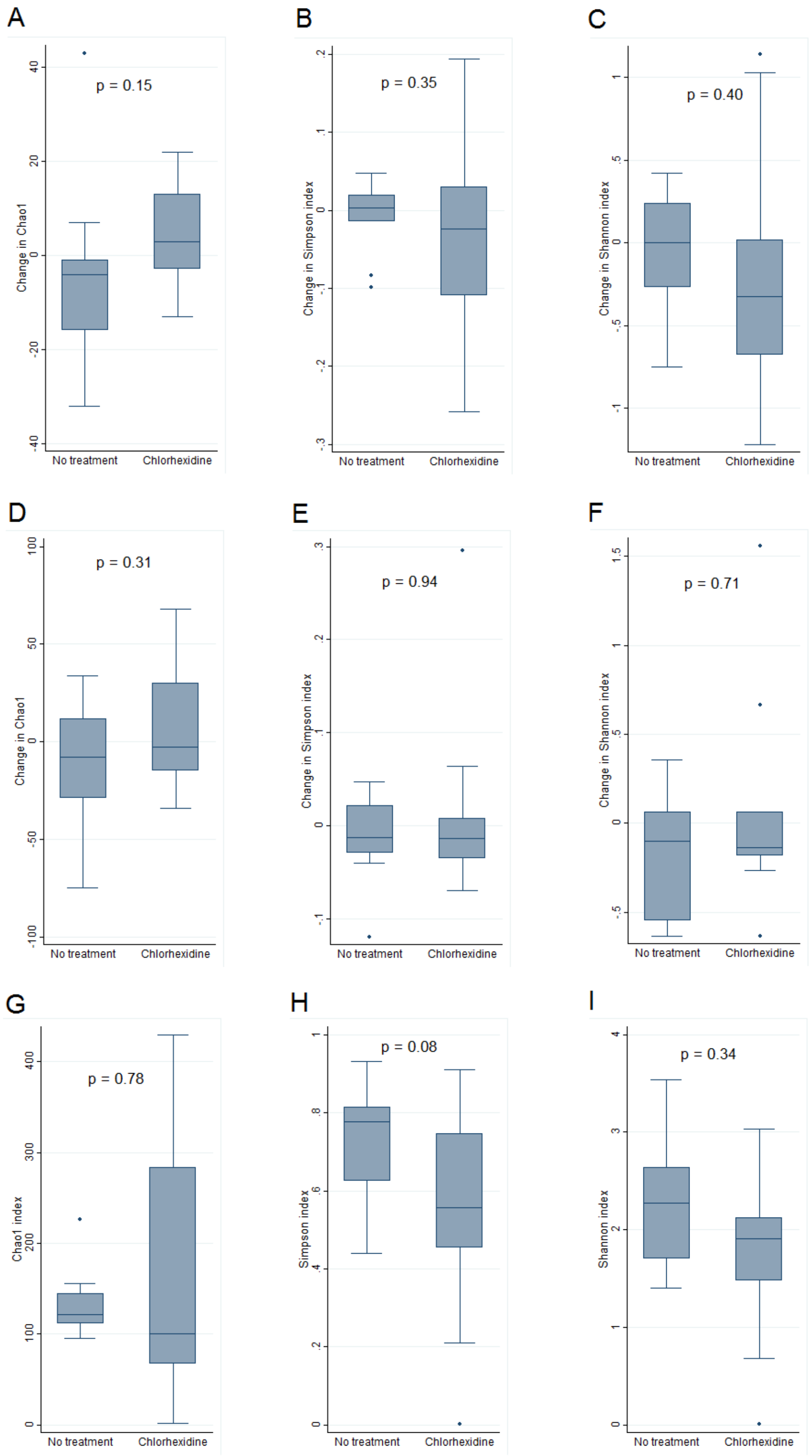

Supplementary Figure 2. There were no significant differences in α-diversity measures between the chlorhexidine and no treatment arms. Change in α-diversity (post- minus pre-treatment) in oral swabs comparing the two arms: (A) Chao (p=0.15), (B) Simpson (p=0.35), (C) Shannon (p=0.40); Change in α-diversity (post- minus pre-treatment) in saliva comparing the two arms: (D) Chao (p=0.31), (E) Simpson (p=0.94), (F) Shannon (p=0.71); Comparison of α-diversity from esophageal squamous tissue comparing the two arms: (G) Chao (p=0.78), (H) Simpson (p=0.08), (I) Shannon (p=0.34).

Supplementary Figure 3.

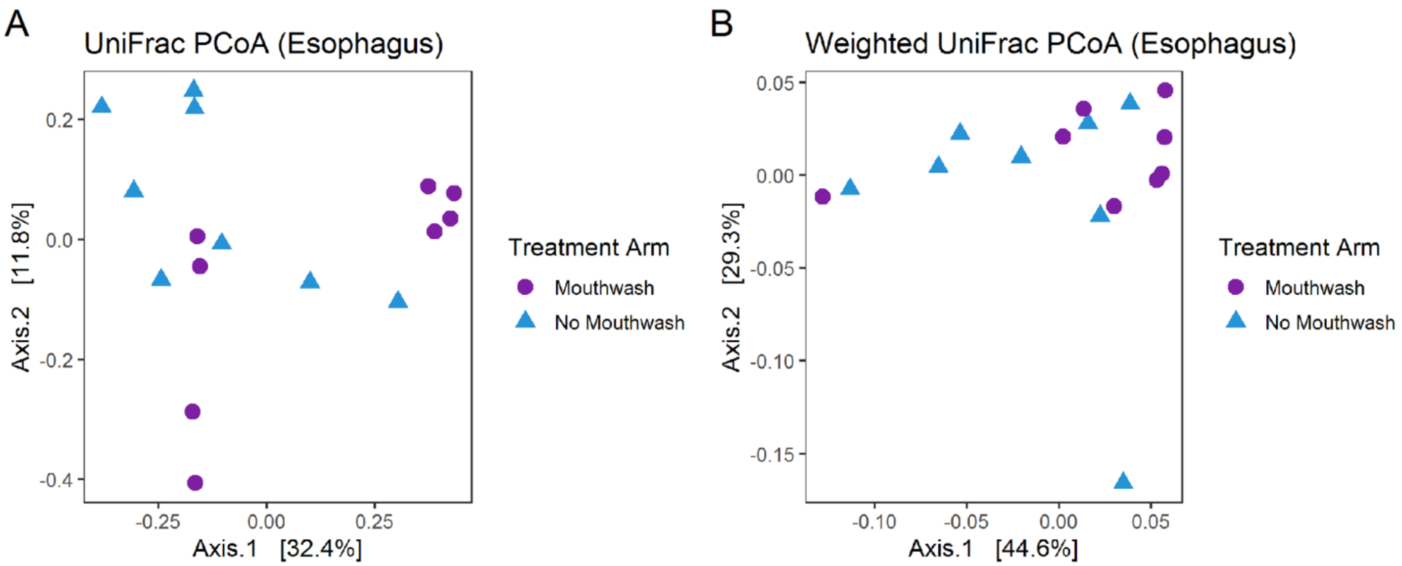

Supplementary Figure 3. In β-diversity analyses, there was a non-significant trend towards a shift in the esophageal microbiome in the chlorhexidine arm (purple circles) as compared to the no treatment arm (light blue triangles): (A) Unweighted principal coordinates analysis (PERMANOVA p=0.08); and (B) weighted principal coordinates analysis (PERMANOVA p=0.07).
