# Supplementary material for: Relationship of the Esophageal Microbiome and Tissue Gene Expression and Links to the Oral Microbiome: A Randomized Clinical Trial
Source: Clin Transl Gastroenterol. 2020 Dec 7;11(12):e00235. doi: 10.14309/ctg.0000000000000235 (PMC7721221; doi:10.14309/ctg.0000000000000235)
Supplement: SUPPLEMENTARY MATERIAL [file ct9-11-e00235-s002.pdf]

Supplementary Table 1. Patient characteristics.

	Chlorhexidine (n=10)	Control (n=10)	p
Age (years)	53.5 (SD 12.9)	51.2 (SD 15.5)	0.75
Sex (male)	4 (40%)	4 (40%)	1.00
Ethnicity/race			0.06
Non-Hispanic, white	2 (20%)	0 (0%)	
Non-Hispanic, non-white	0 (0%)	1 (10%)	
Hispanic, white	4 (40%)	1 (10%)	
Hispanic, non-white	4 (40%)	4 (40%)	
Hispanic, race unknown	0 (0%)	4 (40%)	
PPI use	5 (50%)	5 (50%)	1.00
Aspirin use	4 (40%)	4 (40%)	1.00
Ever smoker	3 (30%)	3 (30%)	1.00
GERD*	7 (70%)	9 (90%)	0.58
BMI (kg/m^2)	29.2 (SD 6.0)	31.2 (SD 5.7)	0.47
WHR	0.94 (SD 0.06)	0.92 (0.08)	0.64
Fat intake, % cal	35.0 (SD 5.6)	33.5 (SD 2.6)	0.43
Fiber intake, gm/day	14.0 (SD 3.8)	13.6 (SD 2.8)	0.81
Indication for EGD			1.00
Reflux symptoms	5	4	
Abdominal pain	2	2	
Dyspepsia	1	2	
Other	2	2	
Endoscopic findings			1.00
Reflux esophagitis	2	3	
Varices	1	0	
Normal esophagus	7	7	
