# Supplementary material for: Relationship of the Esophageal Microbiome and Tissue Gene Expression and Links to the Oral Microbiome: A Randomized Clinical Trial
Source: Clin Transl Gastroenterol. 2020 Dec 7;11(12):e00235. doi: 10.14309/ctg.0000000000000235 (PMC7721221; doi:10.14309/ctg.0000000000000235)
Supplement: SUPPLEMENTARY MATERIAL [file ct9-11-e00235-s003.pdf]

Supplementary Table 2. Baseline oral swab and saliva microbiome composition comparing the chlorhexidine and no treatment arms. The top 20 differentially abundant OTUs (by unadjusted p-value) are listed for oral swabs and for saliva.

Oral swab

	baseM	log2FC	p	padj	Kingdom	Phylum	Class	Order	Family	Genus
Otu29	70.504	4.4753	0.002	0.497	k_Bacteria	p_Proteobacteria	c_Gammaproteobacteria	o_Pasteurellales	f_Pasteurellaceae	g_Haemophilus
Otu225	0.2225	1.1658	0.775	0.999	k_Bacteria	NA	NA	NA	NA	NA
Otu156	0.2579	2.4215	0.55	0.999	k_Bacteria	NA	NA	NA	NA	NA
Otu273	0.1286	-1.256	0.758	0.999	k_Bacteria	NA	NA	NA	NA	NA
Otu178	0.0972	0.8683	0.831	0.999	k_Bacteria	NA	NA	NA	NA	NA
Otu344	0.6953	-2.955	0.465	0.999	k_Bacteria	NA	NA	NA	NA	NA
Otu234	0.099	0.0027	0.999	0.999	k_Bacteria	NA	NA	NA	NA	NA
Otu182	0.2155	0.8683	0.831	0.999	k_Bacteria	NA	NA	NA	NA	NA
Otu367	0.0879	0.0027	0.999	0.999	k_Bacteria	p_Bacteroidetes	NA	NA	NA	NA
Otu333	0.4908	-1.298	0.749	0.999	k_Bacteria	p_Bacteroidetes	NA	NA	NA	NA
Otu212	0.042	0.0027	0.999	0.999	k_Bacteria	p_Bacteroidetes	NA	NA	NA	NA
Otu34	34.025	2.8738	0.041	0.999	k_Bacteria	p_Bacteroidetes	c_Bacteroidia	o_Bacteroidales	f_Prevotellaceae	g_Alloprevotella
Otu215	0.3616	0.0027	0.999	0.999	k_Bacteria	p_Bacteroidetes	c_Bacteroidia	o_Bacteroidales	f_Bacteroidales_incertae_sedis	g_Phocaeicola
Otu54	24.603	-1.413	0.327	0.999	k_Bacteria	p_Bacteroidetes	c_Bacteroidia	o_Bacteroidales	f_Porphyromonadaceae	g_Porphyromonas
Otu646	0.0879	0.0027	0.999	0.999	k_Bacteria	p_Bacteroidetes	NA	NA	NA	NA
Otu534	0.1118	0.8683	0.831	0.999	k_Bacteria	p_Bacteroidetes	NA	NA	NA	NA
Otu490	0.042	0.0027	0.999	0.999	k_Bacteria	p_Bacteroidetes	NA	NA	NA	NA
Otu61	0.2711	-2.251	0.579	0.999	k_Bacteria	p_Bacteroidetes	c_Flavobacteriia	o_Flavobacteriales	f_Flavobacteriaceae	NA
Otu219	1.493	4.9491	0.176	0.999	k_Bacteria	p_Bacteroidetes	c_Flavobacteriia	o_Flavobacteriales	f_Flavobacteriaceae	g_Chryseobacterium
Otu201	1.9454	0.5271	0.836	0.999	k_Bacteria	p_Bacteroidetes	c_Flavobacteriia	o_Flavobacteriales	f_Flavobacteriaceae	NA

Saliva

	baseMe	log2Fol	pvalue	padj	Kingdom	Phylum	Class	Order	Family	Genus
Otu18	144.6	3.1619	3E-04	0.086	k_Bacteria	p_Actinobacteria	c_Actinobacteria	o_Actinomycetales	f_Micrococcaceae	g_Rothia
Otu1	2712	1.5553	0.003	0.449	k_Bacteria	p_Firmicutes	c_Bacilli	o_Lactobacillales	f_Streptococcaceae	g_Streptococcus
Otu68	37.298	3.3512	0.007	0.834	k_Bacteria	p_Fusobacteria	c_Fusobacteriia	o_Fusobacteriales	f_Leptotrichiaceae	g_Leptotrichia
Otu20	604.18	2.9654	0.035	0.999	k_Bacteria	p_Proteobacteria	c_Betaproteobacteria	o_Neisseriales	f_Neisseriaceae	g_Neisseria
Otu89	17.517	-2.621	0.044	0.999	k_Bacteria	p_Bacteroidetes	c_Bacteroidia	o_Bacteroidales	f_Porphyromonadaceae	g_Tannerella
Otu119	11.998	2.9376	0.044	0.999	k_Bacteria	p_Bacteroidetes	c_Bacteroidia	o_Bacteroidales	NA	NA
Otu179	3.8023	-4.964	0.057	0.999	k_Bacteria	p_Firmicutes	c_Clostridia	o_Clostridiales	f_Lachnospiraceae	NA
Otu15	331.82	1.2834	0.059	0.999	k_Bacteria	p_Firmicutes	c_Bacilli	o_Bacillales	f_Bacillales_Incertae_Sedis_XI	g_Gemella
Otu253	4.3581	4.7808	0.068	0.999	k_Bacteria	p_Firmicutes	c_Negativicutes	o_Selenomonadales	f_Veillonellaceae	NA
Otu14	88.898	2.3376	0.07	0.999	k_Bacteria	p_Proteobacteria	c_Gammaproteobacteria	o_Pasteurellales	f_Pasteurellaceae	NA
Otu111	32.882	1.8325	0.095	0.999	k_Bacteria	p_Proteobacteria	c_Gammaproteobacteria	o_Pasteurellales	f_Pasteurellaceae	g_Aggregatibacter
Otu72	8.0418	2.6929	0.099	0.999	k_Bacteria	p_Firmicutes	c_Negativicutes	o_Selenomonadales	f_Veillonellaceae	g_Veillonella
Otu17	458.08	-1.831	0.112	0.999	k_Bacteria	p_Bacteroidetes	c_Bacteroidia	o_Bacteroidales	f_Prevotellaceae	g_Prevotella
Otu281	1.3746	-4.111	0.136	0.999	k_Bacteria	p_Firmicutes	c_Clostridia	o_Clostridiales	f_Lachnospiraceae	g_Johnsonella
Otu41	48.468	1.1087	0.156	0.999	k_Bacteria	p_Proteobacteria	c_Epsilonproteobacteria	o_Campylobacterales	f_Campylobacteraceae	g_Campylobacter

Otu155	7.0574	1.7505	0.159	0.999	k	Bacteria	p_Proteobacteria	c_Ganunaproteobacteria	o Enterobacteriales	f Enterobacteriaceae	NA
Otu79	13.852	1.9283	0.16	0.999	k	Bacteria	p_Synergistetes	c_Synergist:ia	o_Synergistales	f_Synergistaceae	g_Fretibacteriurn
Otu27	25.775	3.7969	0.176	0.999	k	Bacteria	p_Bacteroidetes	c Bacteroidia	o Bacteroidales	f Prevotellaceae	g_Prevotella
Otu64	52.694	1.7235	0.185	0.999	k	Bacteria	p_Candidatus_Saccharibа	g_Saccharibacteria_genera_incerta	NA	NA	NA
Otu136	8.0818	2.1018	0.19	0.999	k	Bacteria	p_Fusobacteria	c Fusobacteriia	o Fusobacteriales	f_Leptobichiaceae	g_Leptotrichia
