# Supplementary material for: Relationship of the Esophageal Microbiome and Tissue Gene Expression and Links to the Oral Microbiome: A Randomized Clinical Trial
Source: Clin Transl Gastroenterol. 2020 Dec 7;11(12):e00235. doi: 10.14309/ctg.0000000000000235 (PMC7721221; doi:10.14309/ctg.0000000000000235)
Supplement: SUPPLEMENTARY MATERIAL [file ct9-11-e00235-s004.pdf]

Supplementary Table 3. Within-individual correlations of taxa across sampling sites, comparing esophagus-saliva pairs and esophagus-oral swab pairs. OTUs chosen for analyses are those with a mean relative abundance >1% and with non-zero reads in >50% of samples across the three sites. P-values are adjusted for multiple comparisons using Bonferroni correction. Statistical significance is defined as an adjusted p-value<0.05. Statistically significant correlations are shown in bold.

OTU1 — d:Bacteria,p:Firmicutes,c:Bacilli,o:Lactobacillales,f:Streptococcaceae,g:Streptococcus

	R^2	adj. p
Esophagus-oral swab	0.23	0.60
Esophagus-saliva	0.01	1.00

OTU2 — d:Bacteria,p:Firmicutes,c:Bacilli,o:Lactobacillales,f:Streptococcaceae,g:Streptococcus

	R^2	adj. p
Esophagus-oral swab	0.004	1.00
Esophagus-saliva	0.07	1.00

OTU3 — d:Bacteria,p:Proteobacteria,c:Gammaproteobacteria,o:Pasteurellales,f:Pasteurellaceae,g:Haemophilus

	R^2	adj. p
Esophagus-oral swab	0.00	1.00
Esophagus-saliva	0.002	1.00

OTU4 — d:Bacteria,p:Bacteroidetes,c:Bacteroidia,o:Bacteroidales,f:Prevotellaceae,g:Prevotella

	R^2	adj. p
Esophagus-oral swab	0.09	1.00
Esophagus-saliva	0.07	1.00

OTU5 — d:Bacteria,p:Fusobacteria,c:Fusobacteriia,o:Fusobacteriales,f:Fusobacteriaceae,g:Fusobacterium

	R^2	adj. p
Esophagus-oral swab	**0.77**	**<0.01**
Esophagus-saliva	**0.72**	**<0.01**

OTU6 — d:Bacteria,p:Firmicutes,c:Negativicutes,o:Selenomonadales,f:Veillonellaceae,g:Veillonella

	R^2	adj. p
Esophagus-oral swab	0.04	1.00
Esophagus-saliva	0.10	1.00

OTU7 — d:Bacteria,p:Firmicutes,c:Bacilli,o:Lactobacillales,f:Streptococcaceae,g:Streptococcus

	R^2	adj. p
Esophagus-oral swab	0.11	1.00
Esophagus-saliva	0.08	1.00

OTU8 d:Bacteria,p:Firmicutes,c:Bacilli,o:Lactobacillales,f:Carnobacteriaceae

	R^2	adj. p
Esophagus-oral swab	0.006	1.00
Esophagus-saliva	**0.46**	**0.02**

OTU11 d:Bacteria,p:Bacteroidetes,c:Bacteroidia,o:Bacteroidales,f:Porphyromonadaceae,g:Porphyromonas

	R^2	adj. p
Esophagus-oral swab	**0.43**	**0.04**
Esophagus-saliva	0.31	0.20

OTU14 d:Bacteria,p:Proteobacteria,c:Gammaproteobacteria,o:Pasteurellales,f:Pasteurellaceae

	R^2	adj. p
Esophagus-oral swab	0.33	0.16
Esophagus-saliva	**0.82**	**<0.01**

OTU15 d:Bacteria,p:Firmicutes,c:Bacilli,o:Bacillales,f:Bacillales_Incertae_Sedis_XI,g:Gemella

	R^2	adj. p
Esophagus-oral swab	0.23	0.58
Esophagus-saliva	0.02	1.00

OTU16 d:Bacteria,p:Bacteroidetes,c:Bacteroidia,o:Bacteroidales

	R^2	adj. p
Esophagus-oral swab	**0.59**	**<0.01**
Esophagus-saliva	0.18	1.00

OTU20 d:Bacteria,p:Proteobacteria,c:Betaproteobacteria,o:Neisseriales,f:Neisseriaceae,g:Neisseria

	R^2	adj. p
Esophagus-oral swab	0.24	0.54
Esophagus-saliva	0.16	1.00

OTU21 d:Bacteria,p:Bacteroidetes,c:Bacteroidia,o:Bacteroidales,f:Prevotellaceae,g:Prevotella

	R^2	adj. p
Esophagus-oral swab	0.33	0.17
Esophagus-saliva	**0.68**	**p<0.01**

OTU29 d:Bacteria,p:Proteobacteria,c:Gammaproteobacteria,o:Pasteurellales,f:Pasteurellaceae,g:Haemophilus

	R^2	adj. p
Esophagus-oral swab	0.16	1.00

Esophagus-saliva	0.07	1.00

OTU44 d:Bacteria,p:Actinobacteria,c:Actinobacteria,o:Actinomycetales,f:Actinomycetaceae,g:Actinomyces

	R^2	adj. p
Esophagus-oral swab	0.04	1.00
Esophagus-saliva	0.08	1.00
