# Supplementary material for: Relationship of the Esophageal Microbiome and Tissue Gene Expression and Links to the Oral Microbiome: A Randomized Clinical Trial
Source: Clin Transl Gastroenterol. 2020 Dec 7;11(12):e00235. doi: 10.14309/ctg.0000000000000235 (PMC7721221; doi:10.14309/ctg.0000000000000235)
Supplement: SUPPLEMENTARY MATERIAL [file ct9-11-e00235-s005.pdf]

Supplementary Table 4. List of significantly altered OTUs in saliva, oral swabs, and esophageal squamous samples, comparing the chlorhexidine and no treatment arms at the end of the study period.

OTUID	Kindgom	Phylum	Class	Order	Family	Genus
Otu72	k_Bacteria	p_Firmicutes	c_Negativicutes	o_Selenomonadales	f_Veillonellaceae	g_Veillonella
Otu31	k_Bacteria	p_SR1	g_SR1_genera_incertae_sedis	NA	NA	NA
Otu29	k_Bacteria	p_Proteobacteria	c_Gammaproteobacteria	o_Pasteurellales	f_Pasteurellaceae	g_Haemophilus
Otu13	k_Bacteria	p_Bacteroidetes	c_Bacteroidia	o_Bacteroidales	NA	NA
Otu483	k_Bacteria	NA	NA	NA	NA	NA
Otu65	k_Bacteria	p_Proteobacteria	c_Gammaproteobacteria	o_Pasteurellales	f_Pasteurellaceae	NA
Otu28	k_Bacteria	p_Bacteroidetes	c_Bacteroidia	o_Bacteroidales	f_Prevotellaceae	g_Prevotella
Otu3	k_Bacteria	p_Proteobacteria	c_Gammaproteobacteria	o_Pasteurellales	f_Pasteurellaceae	g_Haemophilus
Otu73	k_Bacteria	p_Actinobacteria	c_Actinobacteria	o_Actinomycetales	f_Corynebacteriaceae	g_Corynebacterium
Otu250	k_Bacteria	NA	NA	NA	NA	NA
Otu315	k_Bacteria	NA	NA	NA	NA	NA
Otu654	k_Bacteria	p_Proteobacteria	c_Gammaproteobacteria	o_Pasteurellales	f_Pasteurellaceae	g_Haemophilus
Otu172	k_Bacteria	p_Firmicutes	c_Bacilli	o_Bacillales	f_Bacillales_Incertae_Sedis_XI	g_Gemella
Otu11	k_Bacteria	p_Bacteroidetes	c_Bacteroidia	o_Bacteroidales	f_Porphyromonadaceae	g_Porphyromonas
Otu71	k_Bacteria	p_Actinobacteria	c_Actinobacteria	o_Actinomycetales	f_Actinomycetaceae	g_Actinomyces
Otu422	k_Bacteria	NA	NA	NA	NA	NA
Otu125	k_Bacteria	p_Firmicutes	c_Clostridia	o_Clostridiales	f_Peptococcaceae_1	g_Peptococcus
Otu271	k_Bacteria	NA	NA	NA	NA	NA
Otu26	k_Bacteria	p_Proteobacteria	c_Epsilonproteobacteria	o_Campylobacterales	f_Campylobacteraceae	g_Campylobacter
Otu64	k_Bacteria	p_Candidatus_Saccharibacteria	g_Saccharibacteria_genera_incertae_sedis	NA	NA	NA
Otu32	k_Bacteria	p_SR1	g_SR1_genera_incertae_sedis	NA	NA	NA
Otu380	k_Bacteria	p_Bacteroidetes	c_Flavobacteriia	o_Flavobacteriales	f_Flavobacteriaceae	g_Capnocytophaga
Otu18	k_Bacteria	p_Actinobacteria	c_Actinobacteria	o_Actinomycetales	f_Micrococcaceae	g_Rothia
Otu10	k_Bacteria	p_Actinobacteria	c_Actinobacteria	o_Actinomycetales	f_Micrococcaceae	g_Rothia
Otu53	k_Bacteria	p_Bacteroidetes	c_Flavobacteriia	o_Flavobacteriales	f_Flavobacteriaceae	g_Capnocytophaga
Otu88	k_Bacteria	p_Proteobacteria	c_Betaproteobacteria	o_Neisseriales	f_Neisseriaceae	g_Eikenella
Otu4	k_Bacteria	p_Bacteroidetes	c_Bacteroidia	o_Bacteroidales	f_Prevotellaceae	g_Prevotella

Esophagus				***Oral Swabs***				***Saliva***			
baseMean	log2FoldChar	pvalue	padj	baseMean	log2FoldChar	pvalue	padj	baseMean	log2FoldChar	pvalue	padj
24.6644111	-7.0110595	**8.22E-06**	**0.00567657**	16.2078558	-4.0541126	**0.04799691**	0.98690644	8.04183851	-0.8421356	0.60535851	0.99860012
43.7657373	-8.8282045	**5.07E-05**	**0.01752951**	45.2457458	-3.716401	**0.08123624**	0.98690644	173.479224	-0.4243722	0.84726865	0.99860012
278.945013	-4.2304552	**0.00018509**	**0.04263185**	70.5041431	-3.0506406	**0.04252323**	0.98690644	58.6423769	-1.5487125	0.16201861	0.99860012
75.7109819	-4.8774669	**0.00091919**	0.15879031	112.537885	-0.9908121	0.53385965	0.98690644	98.5701142	-2.0210652	0.14370861	0.99860012
2.76544361	5.01879404	**0.00484763**	0.5866415	0.17895608	-2.1045726	0.62338125	0.98690644	0.09588736	0.97974678	0.8143873	0.99860012
8.33214344	-5.1132281	**0.0054678**	0.5866415	5.6159245	-4.3676012	0.06494895	0.98690644	22.8228242	-2.461594	0.13226524	0.99860012
7.59823465	-4.6834161	**0.00594282**	0.5866415	10.9415109	-2.7400481	0.14437638	0.98690644	91.412019	-2.1591501	0.09696285	0.99860012
421.890392	-2.5752873	**0.00924488**	0.79852668	1608.6395	-0.8663752	0.3219623	0.98690644	3797.4612	-1.0704104	0.17388777	0.99860012
2.92514199	-4.9239775	**0.01042739**	0.80059169	0.76456881	1.2113675	0.73671106	0.98690644	61.9960213	-2.1920088	0.05695715	0.99860012
2.37706194	4.79996214	**0.01455138**	0.8594949	NA	NA	NA	NA	NA	NA	NA	NA
3.35896433	4.1548646	**0.02522367**	0.8594949	NA	NA	NA	NA	0.06880783	0.66249977	0.8740113	0.99860012
30.6465551	-5.2343613	**0.02656672**	0.8594949	9.51507423	-1.957091	0.24470202	0.98690644	22.8702036	-2.1430564	0.14405599	0.99860012
8.69969421	-6.49631	**0.02880294**	0.8594949	NA	NA	NA	NA	NA	NA	NA	NA
146.279588	-2.340089	**0.03287846**	0.8594949	337.830491	-1.8840391	0.1288242	0.98690644	1164.96234	-0.7526105	0.44411865	0.99860012
23.8904659	-2.5357787	**0.03532464**	0.8594949	6.62543383	-0.0608706	0.9628202	0.98690644	74.6749181	-0.8273011	0.46091927	0.99860012
2.06749872	4.59826084	**0.03549043**	0.8594949	NA	NA	NA	NA	NA	NA	NA	NA
4.17422188	-4.1489479	**0.03562255**	0.8594949	0.96273473	0.31052794	0.9225562	0.98690644	7.42901918	-0.755299	0.61194701	0.99860012
1.85803381	4.44523522	**0.0429684**	0.8594949	NA	NA	NA	NA	0.25640819	-1.2933313	0.75633816	0.99860012
28.0791262	-1.8561907	**0.04306628**	0.8594949	82.1072019	-0.5919487	0.53159106	0.98690644	136.116493	-1.6798079	0.05491782	0.99860012
1.90865362	-4.3192891	**0.04689952**	0.8594949	18.2542758	-1.3695229	0.36497852	0.98690644	52.6944564	-0.2655276	0.83877971	0.99860012
13.3984113	-5.8384425	**0.04898449**	0.8594949	3.39776788	0.21203748	0.91895698	0.98690644	37.9296264	-2.1071348	0.30583717	0.99860012
1.09113274	2.02587688	0.49857207	0.8594949	30.0031802	7.75065633	**9.68E-07**	**0.00027388**	26.9750163	3.29574216	**0.00581122**	0.90680107
29.6095153	-1.7063948	0.18328771	0.8594949	83.5509778	3.97068784	**0.00333277**	0.47158693	144.60461	-0.7404059	0.39258702	0.99860012
310.688793	0.58540361	0.57456431	0.8594949	338.769595	-1.8152718	**0.01270724**	0.86109964	1821.3634	-0.7646599	0.23758588	0.99860012
36.4118461	1.28476246	0.30423111	0.8594949	75.2813032	2.90162307	**0.013918**	0.86109964	89.1273213	0.93093704	0.32132717	0.99860012
5.32066804	1.26259654	0.36702759	0.8594949	5.39631138	4.32513001	**0.01521377**	0.86109964	47.7558438	0.88464318	0.26706973	0.99860012
119.286838	-1.6535587	0.18957545	0.8594949	439.385287	-2.4368143	**0.03274427**	0.98690644	2355.41207	-1.0272366	0.31208279	0.99860012
