# Supplementary material for: Relationship of the Esophageal Microbiome and Tissue Gene Expression and Links to the Oral Microbiome: A Randomized Clinical Trial
Source: Clin Transl Gastroenterol. 2020 Dec 7;11(12):e00235. doi: 10.14309/ctg.0000000000000235 (PMC7721221; doi:10.14309/ctg.0000000000000235)
Supplement: SUPPLEMENTARY MATERIAL [file ct9-11-e00235-s006.pdf]

**Supplementary Table 5.** List of genes with significantly altered expression in squamous esophagus, comparing the chlorhexidine arm to the no treatment arm.

| Gene | log2Fold | pvalue | padj | Gene | log2Fold | pvalue | padj | Gene | log2Fold | pvalue | padj |
|---|---|---|---|---|---|---|---|---|---|---|---|
| LIPF | -8.8042 | 2.93E-08 | 0.00025 | APOE | -2.21011 | 0.0003 | 0.05204 | TUBA8 | -1.7106 | 0.0009 | 0.08303 |
| KCNE2 | -8.5422 | 1.87E-08 | 0.00025 | LCN10 | -2.13546 | 0.0003 | 0.05204 | HES6 | -1.6926 | 0.0009 | 0.08303 |
| POSTN | -4.752 | 6.04E-08 | 0.00034 | ZNF579 | -1.97365 | 0.0003 | 0.05204 | LHFPL2 | -1.6813 | 0.0009 | 0.08303 |
| PGC | -8.8499 | 1.20E-07 | 0.00041 | ANPEP | -1.97144 | 0.0003 | 0.05204 | APOL1 | -1.6789 | 0.0009 | 0.08303 |
| GHRL | -6.0059 | 9.84E-08 | 0.00041 | CD1D | -1.93763 | 0.0003 | 0.05204 | HEXDC | -1.4602 | 0.0009 | 0.08303 |
| HDC | -4.46 | 1.58E-07 | 0.00045 | C16orf79 | -1.82674 | 0.0003 | 0.05204 | NPPA-AS1 | -1.3295 | 0.0009 | 0.08303 |
| PGA5 | -8.495 | 5.08E-07 | 0.00124 | FUT1 | -1.81591 | 0.0003 | 0.05204 | CORO7 | -1.2828 | 0.0009 | 0.08303 |
| MZB1 | -4.9369 | 5.79E-07 | 0.00124 | TMEM86B | -1.75523 | 0.0003 | 0.05204 | C10orf125 | -1.0848 | 0.0009 | 0.08303 |
| CBS | -5.6598 | 9.87E-07 | 0.00187 | NES | -1.41118 | 0.0003 | 0.05204 | GRASP | -1.3627 | 0.0009 | 0.08308 |
| ERN2 | -6.1235 | 1.13E-06 | 0.00194 | TIMP1 | -1.83136 | 0.0003 | 0.05233 | KIF19 | -2.5143 | 0.001 | 0.08441 |
| PSAPL1 | -4.0785 | 1.31E-06 | 0.00203 | MC1R | -1.60343 | 0.0003 | 0.05301 | AATK | -2.3463 | 0.001 | 0.08441 |
| PGA3 | -9.9099 | 1.52E-06 | 0.00216 | SYT5 | -5.40653 | 0.0003 | 0.05588 | TMEM88 | -1.9721 | 0.001 | 0.08441 |
| C4orf48 | -3.7665 | 1.77E-06 | 0.00233 | IGLL5 | -4.08305 | 0.0003 | 0.05588 | FAM176B | -1.5129 | 0.001 | 0.08441 |
| CLDN18 | -8.0104 | 2.06E-06 | 0.00235 | BEX1 | -4.84812 | 0.0004 | 0.05634 | SLC43A2 | -1.4965 | 0.001 | 0.08441 |
| CREB3L1 | -4.4703 | 2.03E-06 | 0.00235 | SELL | -1.76646 | 0.0004 | 0.05634 | NR4A1 | -1.4516 | 0.001 | 0.08441 |
| COL2A1 | -8.2628 | 3.41E-06 | 0.00353 | MBL1P | -2.61023 | 0.0004 | 0.05732 | MUC5AC | -4.6882 | 0.0011 | 0.08558 |
| APLP1 | -4.0123 | 3.51E-06 | 0.00353 | FAM195A | -1.80425 | 0.0004 | 0.05734 | CTSE | -4.1796 | 0.0011 | 0.08558 |
| CAPN13 | -2.8996 | 3.99E-06 | 0.00378 | C12orf28 | -3.02994 | 0.0004 | 0.05753 | WFDC3 | -2.7802 | 0.001 | 0.08558 |
| C10orf92 | -6.6971 | 4.46E-06 | 0.00401 | EMILIN2 | -1.76457 | 0.0004 | 0.05753 | EGR2 | -2.2503 | 0.001 | 0.08558 |
| PDIA2 | -6.3495 | 5.99E-06 | 0.00512 | DUSP15 | -2.57121 | 0.0004 | 0.05888 | FEZ1 | -1.9869 | 0.0011 | 0.08558 |
| HEPACAM2 | -4.8266 | 6.62E-06 | 0.00539 | SERPINA1 | -2.29245 | 0.0004 | 0.05963 | HSD17B6 | -1.8714 | 0.0011 | 0.08558 |
| LYPD6B | -3.2956 | 8.77E-06 | 0.00681 | EMID1 | -2.92726 | 0.0004 | 0.05992 | PDE9A | -1.8064 | 0.0011 | 0.08558 |
| PIGR | -4.9667 | 9.88E-06 | 0.00733 | TMEM176A | -2.60118 | 0.0004 | 0.05992 | IGFBP3 | -1.5993 | 0.001 | 0.08558 |
| AZGP1 | -6.3972 | 1.37E-05 | 0.00974 | FAM155B | -2.7948 | 0.0004 | 0.06093 | PMEPA1 | -1.5934 | 0.001 | 0.08558 |
| SIX2 | -5.5658 | 1.72E-05 | 0.01133 | FLJ33360 | -2.89444 | 0.0004 | 0.06107 | SLC22A1 | -1.5629 | 0.0011 | 0.08558 |
| ADAMTS15 | -3.5183 | 1.66E-05 | 0.01133 | TMEM47 | -1.4411 | 0.0004 | 0.06107 | RASSF7 | -1.4939 | 0.0011 | 0.08558 |
| SPOCD1 | -5.7944 | 1.80E-05 | 0.01141 | CD72 | -1.9179 | 0.0004 | 0.06166 | BATF3 | -1.425 | 0.001 | 0.08558 |
| CXCL13 | -6.0486 | 2.63E-05 | 0.015 | HYAL1 | -1.84907 | 0.0004 | 0.06166 | LOC113230 | -1.7477 | 0.0011 | 0.08605 |
| TFF2 | -5.7025 | 2.56E-05 | 0.015 | SLC5A5 | -4.2351 | 0.0004 | 0.06169 | NAT14 | -1.4917 | 0.0011 | 0.08723 |
| TMED6 | -4.2786 | 2.61E-05 | 0.015 | CNTD1 | -4.13432 | 0.0005 | 0.06343 | KC6 | 2.11741 | 0.0011 | 0.08723 |
| LOC10013088 | 1.49239 | 2.91E-05 | 0.01604 | MUC6 | -5.82478 | 0.0005 | 0.0643 | SYNPO | -1.2827 | 0.0011 | 0.08827 |
| HPN | -6.0715 | 3.11E-05 | 0.01662 | CCDC24 | -1.7936 | 0.0005 | 0.06436 | IGFALS | -5.0004 | 0.0011 | 0.08829 |
| CPA2 | -8.8774 | 4.14E-05 | 0.02145 | CPAMD8 | -2.62293 | 0.0005 | 0.06506 | SLC43A1 | -2.3026 | 0.0011 | 0.08829 |
| UBE2QL1 | -4.8361 | 4.53E-05 | 0.02221 | LGALS9 | -1.92076 | 0.0005 | 0.06506 | PGAM2 | -1.4864 | 0.0011 | 0.08829 |
| SLC45A4 | -1.4673 | 4.55E-05 | 0.02221 | REG1A | -7.41866 | 0.0005 | 0.06596 | AMH | -1.2003 | 0.0011 | 0.08829 |
| SHC2 | -2.9277 | 5.36E-05 | 0.02475 | CLCNKA | -5.09023 | 0.0005 | 0.06596 | OOEP | -2.7058 | 0.0012 | 0.08972 |
| PPP2R2B | 1.48282 | 5.24E-05 | 0.02475 | CAPN8 | -4.11824 | 0.0005 | 0.06596 | LGALS4 | -2.5747 | 0.0012 | 0.08972 |
| MS4A1 | -3.5032 | 6.32E-05 | 0.02839 | C17orf110 | -3.85579 | 0.0005 | 0.06596 | INPP5J | -1.6506 | 0.0012 | 0.08972 |
| KCNH2 | -3.2848 | 6.72E-05 | 0.02927 | GPC3 | -2.7803 | 0.0005 | 0.06596 | ZGLP1 | -1.2911 | 0.0012 | 0.08972 |
| TSPAN15 | -2.4199 | 6.87E-05 | 0.02927 | TNNC1 | -2.63673 | 0.0005 | 0.06596 | PRKD1 | -1.6057 | 0.0012 | 0.08998 |
| CADPS2 | -1.7345 | 7.03E-05 | 0.02927 | LOC115110 | -2.33429 | 0.0005 | 0.06596 | LOC145837 | -3.5305 | 0.0012 | 0.09005 |
| LINGO4 | -2.9099 | 7.58E-05 | 0.03082 | C3orf32 | -2.2914 | 0.0005 | 0.06596 | TNFRSF8 | -2.6757 | 0.0012 | 0.09011 |
| VSIG1 | -3.7325 | 8.31E-05 | 0.03302 | FCGR3A | -2.0374 | 0.0005 | 0.06596 | ZNF444 | -1.5987 | 0.0012 | 0.09011 |
| LINC00092 | -4.4059 | 9.57E-05 | 0.03715 | TMEM125 | -1.79921 | 0.0005 | 0.06596 | ATP6V0E2 | -1.2815 | 0.0012 | 0.09011 |
| SH3GL2 | -5.9772 | 0.0001009 | 0.03746 | RASIP1 | -1.75681 | 0.0005 | 0.06596 | LOC1005061 | -4.1753 | 0.0012 | 0.09035 |
| ASPHD1 | -3.3728 | 9.90E-05 | 0.03746 | ARVCF | -1.69395 | 0.0005 | 0.06596 | ITPKA | -2.7939 | 0.0012 | 0.09035 |
| GKN2 | -4.9336 | 0.0001036 | 0.03765 | CALML6 | -1.6971 | 0.0006 | 0.0663 | PODXL2 | -1.5398 | 0.0013 | 0.09203 |
| SDK1 | -1.4821 | 0.000107 | 0.03809 | MATN4 | -2.18907 | 0.0006 | 0.06805 | TMEM198 | -1.5145 | 0.0013 | 0.09222 |
| WBSCR17 | -3.0478 | 0.000112 | 0.0389 | TBXA2R | -2.03212 | 0.0006 | 0.06805 | CXCR5 | -3.4049 | 0.0013 | 0.09262 |
| PLIN5 | -2.8131 | 0.0001184 | 0.0389 | EIF5A2 | -1.87547 | 0.0006 | 0.06805 | KCNJ11 | -2.4618 | 0.0013 | 0.09355 |
| GADD45G | -2.2797 | 0.000114 | 0.0389 | TCIRG1 | -1.39402 | 0.0006 | 0.06805 | MAGIX | -1.5766 | 0.0013 | 0.09355 |
| GLYCTK | -2.159 | 0.0001172 | 0.0389 | TNFRSF18 | -1.39262 | 0.0006 | 0.06805 | POU5F1 | -2.1984 | 0.0013 | 0.094 |
| C16orf89 | -6.0241 | 0.0001231 | 0.03899 | GRK5 | -1.78772 | 0.0006 | 0.0693 | DAAM2 | -2.0977 | 0.0013 | 0.094 |
| FER1L4 | -3.3538 | 0.0001233 | 0.03899 | C21orf2 | -1.66843 | 0.0006 | 0.0693 | LOC1005058 | -1.3997 | 0.0013 | 0.094 |
| KCNQ1 | -3.0936 | 0.0001405 | 0.04209 | NEK6 | -1.42963 | 0.0006 | 0.07017 | CLCN4 | -1.3767 | 0.0013 | 0.094 |

| | | | | | | | | | | | |
|---|---|---|---|---|---|---|---|---|---|---|---|
| BCL2L15 | -2.2967 | 0.0001385 | 0.04209 | FAM20C | -1.54871 | 0.0006 | 0.07024 | CDHR2 | -2.9873 | 0.0013 | 0.09455 |
| C1QTNF1 | -2.2356 | 0.0001491 | 0.04391 | EPPK1 | -1.80151 | 0.0006 | 0.07072 | CCDC107 | -1.5791 | 0.0013 | 0.09455 |
| PP7080 | -1.994 | 0.0001553 | 0.04438 | TFF1 | -4.49422 | 0.0007 | 0.07216 | C9orf142 | -1.1589 | 0.0014 | 0.09455 |
| UCP2 | -1.6256 | 0.0001559 | 0.04438 | DUSP26 | -3.83233 | 0.0007 | 0.07216 | HLA-J | -2.0202 | 0.0014 | 0.09473 |
| SLC26A9 | -3.8943 | 0.0001704 | 0.04695 | TMEM139 | -2.11584 | 0.0007 | 0.07216 | TNFRSF14 | -1.5335 | 0.0014 | 0.09473 |
| SCAMP5 | 1.6776 | 0.0001684 | 0.04695 | MDK | -1.86051 | 0.0007 | 0.07216 | MSLNL | -6.8108 | 0.0014 | 0.09486 |
| EXOC3L4 | -3.5383 | 0.0001863 | 0.04812 | SEZ6L2 | -2.64124 | 0.0007 | 0.07487 | FCRL2 | -2.9924 | 0.0014 | 0.09553 |
| CXCL1 | -3.3405 | 0.0001887 | 0.04812 | C2CD4C | -2.39409 | 0.0007 | 0.07487 | LINC00085 | -1.1865 | 0.0014 | 0.09553 |
| GNAZ | -3.0089 | 0.0001875 | 0.04812 | LPL | -1.78321 | 0.0007 | 0.07487 | SLC44A4 | -2.9551 | 0.0014 | 0.09561 |
| PTGIR | -2.5461 | 0.000191 | 0.04812 | CAPS | -1.75641 | 0.0007 | 0.07784 | FA2H | -1.9641 | 0.0014 | 0.09561 |
| FN3K | -2.365 | 0.0001916 | 0.04812 | DIO3OS | -4.14856 | 0.0008 | 0.07877 | TP53I13 | -1.3778 | 0.0014 | 0.09561 |
| APLN | -1.8842 | 0.0001876 | 0.04812 | ARSE | -3.25667 | 0.0007 | 0.07877 | PLIN4 | -1.6489 | 0.0014 | 0.09693 |
| CHIA | -9.2441 | 0.0002184 | 0.05138 | LCN12 | -2.23732 | 0.0008 | 0.07877 | PEBP4 | -5.9467 | 0.0015 | 0.09858 |
| PHGR1 | -5.0729 | 0.0002259 | 0.05138 | SDSL | -2.04578 | 0.0007 | 0.07877 | KCNE4 | -3.1842 | 0.0015 | 0.09858 |
| GKN1 | -4.2499 | 0.0002346 | 0.05138 | TESC | -2.92143 | 0.0008 | 0.08114 | C19orf45 | -2.8341 | 0.0015 | 0.09858 |
| NFE2 | -3.6122 | 0.0002276 | 0.05138 | RENBP | -1.79366 | 0.0008 | 0.08114 | KCNJ8 | -2.3869 | 0.0015 | 0.09858 |
| LOC10050550 | -3.1102 | 0.0002302 | 0.05138 | FZD8 | -1.74154 | 0.0008 | 0.08114 | HMGA1P4 | -2.1505 | 0.0015 | 0.09858 |
| SCG5 | -2.5206 | 0.0002337 | 0.05138 | PRR15L | -1.66704 | 0.0008 | 0.08114 | PLCE1 | -2.0185 | 0.0015 | 0.09858 |
| TNFRSF4 | -2.3101 | 0.0002093 | 0.05138 | TNFRSF17 | -3.9619 | 0.0008 | 0.08137 | LOC643719 | -2.0115 | 0.0015 | 0.09858 |
| C9orf150 | -2.1675 | 0.0002136 | 0.05138 | TSPAN8 | -3.54964 | 0.0008 | 0.08137 | CSPG4 | -2.0048 | 0.0015 | 0.09858 |
| LOC10012807 | -1.7439 | 0.0002292 | 0.05138 | CAMK2B | -2.17816 | 0.0008 | 0.08137 | FAM110B | -1.9506 | 0.0015 | 0.09858 |
| G0S2 | -2.6206 | 0.0002404 | 0.05146 | FAM167B | -2.01061 | 0.0008 | 0.08137 | DGKD | -1.9426 | 0.0015 | 0.09858 |
| COL4A3 | -2.4714 | 0.000241 | 0.05146 | DIRAS1 | -1.99112 | 0.0008 | 0.08137 | CPNE7 | -1.6708 | 0.0015 | 0.09858 |
| CHGA | -5.6165 | 0.0002444 | 0.05153 | PARVB | -1.63659 | 0.0008 | 0.08137 | ZNF653 | -0.9785 | 0.0016 | 0.09974 |
| LINC00261 | -7.2677 | 0.0002827 | 0.05204 | LOC200772 | -5.69171 | 0.0009 | 0.08303 | CD81 | -0.8307 | 0.0005 | 0.06596 |
| B3GAT1 | -3.9128 | 0.0002919 | 0.05204 | INSRR | -4.86905 | 0.0009 | 0.08303 | HERC2P9 | -0.7941 | 0.0001 | 0.04008 |
| SULT1A2 | -3.8786 | 0.0002882 | 0.05204 | KIAA0125 | -3.41596 | 0.0009 | 0.08303 | STAC3 | -0.7805 | 0.0003 | 0.05469 |
| AGAP1-IT1 | -3.4194 | 0.0003049 | 0.05204 | CCL19 | -2.80432 | 0.0009 | 0.08303 | LOC143188 | -0.7776 | 0.0015 | 0.09858 |
| HK3 | -2.9668 | 0.0002617 | 0.05204 | SLC29A4 | -2.58427 | 0.0009 | 0.08303 | ORAI2 | -0.7183 | 0.0002 | 0.05138 |
| ASRGL1 | -2.8574 | 0.0002649 | 0.05204 | VPREB3 | -2.38049 | 0.0009 | 0.08303 | LMO4 | -0.4964 | 0.0005 | 0.06596 |
| TAGLN | -2.8522 | 0.0003027 | 0.05204 | PLAC8L1 | -2.21846 | 0.0009 | 0.08303 | NXN | 0.28617 | 0.0009 | 0.08303 |
| FBP1 | -2.7637 | 0.0002865 | 0.05204 | TMEM178 | -2.01141 | 0.0009 | 0.08303 | MTMR9LP | -1.6067 | 0.0015 | 0.09858 |
| PKDCC | -2.7259 | 0.0002926 | 0.05204 | TCAP | -1.88988 | 0.0009 | 0.08303 | C9orf7 | -1.5548 | 0.0015 | 0.09858 |
| HSH2D | -2.4078 | 0.0002882 | 0.05204 | LGALS1 | -1.845 | 0.0009 | 0.08303 | DPM2 | -1.2984 | 0.0015 | 0.09858 |
| LRRC4B | -2.2672 | 0.0002786 | 0.05204 | MAPK11 | -1.72207 | 0.0009 | 0.08303 | C2CD4A | -3.8319 | 0.0016 | 0.09974 |
