# Supplementary material for: Relationship of the Esophageal Microbiome and Tissue Gene Expression and Links to the Oral Microbiome: A Randomized Clinical Trial
Source: Clin Transl Gastroenterol. 2020 Dec 7;11(12):e00235. doi: 10.14309/ctg.0000000000000235 (PMC7721221; doi:10.14309/ctg.0000000000000235)
Supplement: SUPPLEMENTARY MATERIAL [file ct9-11-e00235-s007.pdf]

Supplementary Table 6. List of genes with significantly altered expression in squamous esophagus, comparing subjects with high (above the median) versus low (below the median) relative abundance of OTU29 in the esophagus. Genes also differentially expressed based on treatment arm are highlighted in light purple.

Gene	log2Fold	pvalue	padj	Gene	log2Fold	pvalue	padj	Gene	log2Fold	pvalue	padj
SERPINA5	22.998	1.08E-14	1.88E-10	FLJ33360	3.069887	0.0006	0.05123	SLC43A1	2.557699	0.00188	0.08249
KRT1	-3.9637	9.37E-10	8.15E-06	MAGIX	1.835505	0.0006	0.05123	ACAP3	0.627818	0.00189	0.08271
HDC	5.1675	7.99E-09	4.63E-05	ARFGAP1	1.531941	0.0006	0.05223	CD72	1.988404	0.00193	0.08359
LIPF	9.0916	1.08E-08	4.68E-05	ATXN7L2	0.71052	0.0006	0.05223	HLA-J	2.177034	0.00194	0.08359
PGC	8.8174	9.00E-08	0.000313	BCL2L15	2.372011	0.0006	0.05223	IZUMO1	2.290024	0.00194	0.08359
KCNE2	8.296	3.53E-07	0.000767	CCDC24	1.979338	0.0006	0.05223	NELF	1.088849	0.00195	0.08359
MZB1	5.4593	3.24E-07	0.000767	FAM195A	1.967555	0.0006	0.05223	PHKG2	1.022148	0.00194	0.08359
PHGR1	5.8897	2.77E-07	0.000767	SCG5	2.648305	0.0006	0.05223	PPP1R16A	1.343538	0.00192	0.08359
PDIA2	7.0322	4.50E-07	0.000869	CRISPLD2	1.905707	0.0006	0.0527	RASGRP4	0.958369	0.00192	0.08359
GHRL	6.4043	6.15E-07	0.001069	RERGL	3.691768	0.0006	0.0527	SELL	1.814737	0.00194	0.08359
LOC729162	-6.5235	8.60E-07	0.00136	MTG1	1.009464	0.0006	0.05292	CD1D	1.900372	0.00196	0.08386
PGA3	9.6996	1.25E-06	0.001808	NEK6	1.572362	0.0006	0.05292	WASH1	1.378828	0.00196	0.08386
POSTN	4.807	1.49E-06	0.001991	BTK	1.018221	0.0006	0.05318	MICAL1	1.15193	0.00197	0.08386
SLC5A5	5.1689	1.83E-06	0.002275	KLKB1	3.208012	0.0007	0.05369	PTGIR	2.399189	0.00201	0.08531
CNTFR	3.8088	2.03E-06	0.002359	DAAM2	2.457016	0.0007	0.05383	LOC339352	1.953527	0.00201	0.08544
MS4A1	4.4488	2.68E-06	0.002909	TMEM88	2.270472	0.0007	0.0549	FRZB	2.121515	0.00202	0.0855
CBS	5.9247	2.95E-06	0.003017	CARNS1	3.459978	0.0007	0.05495	TRABD	0.926981	0.00203	0.0855
CD22	2.8144	4.53E-06	0.004303	RASSF4	1.774211	0.0007	0.05509	CCDC68	1.940245	0.00204	0.08565
DERL3	3.9829	4.95E-06	0.004303	FCRL1	3.523247	0.0007	0.0552	SCN1B	2.26448	0.00204	0.08565
WBSCR17	3.8109	4.76E-06	0.004303	MSLN	3.218157	0.0007	0.05601	SOLH	0.565127	0.00204	0.08565
GKN2	5.4319	5.60E-06	0.004636	MST1P9	2.68898	0.0007	0.05601	APLN	1.552044	0.00207	0.08636
APOE	2.8177	6.66E-06	0.005261	VGF	3.627777	0.0007	0.05601	PVR	0.675234	0.00207	0.08636
CA9	6.315	7.22E-06	0.00543	LOC100505	3.995997	0.0007	0.05622	ACP2	0.983974	0.0021	0.08642
PALM3	5.9101	7.49E-06	0.00543	LOC100507	1.845118	0.0007	0.05832	ATG4B	0.790982	0.00213	0.08642
C4orf48	3.8668	8.45E-06	0.005733	RASL10A	2.271713	0.0008	0.0585	C8G	1.038851	0.0021	0.08642
PGA5	8.5169	8.57E-06	0.005733	RUNDC2C	3.96747	0.0008	0.05919	CAPN6	-1.40867	0.00213	0.08642
FLJ42875	7.4875	1.07E-05	0.00665	ARVCF	1.884078	0.0008	0.06058	FCRL5	3.388929	0.00209	0.08642
SPOCD1	6.2829	1.06E-05	0.00665	DUSP26	4.296819	0.0008	0.06058	IGFALS	5.323341	0.00213	0.08642
ADAMTS15	3.9467	1.18E-05	0.007094	FCRLA	5.088727	0.0008	0.06058	LDLRAD2	2.367528	0.00211	0.08642
PSAPL1	4.2031	1.24E-05	0.007165	GBA2	0.729561	0.0008	0.06058	MIR378I	2.750183	0.00208	0.08642
SPRR2G	-3.5683	1.38E-05	0.007767	KCNE4	3.570066	0.0008	0.06058	PRR15L	1.763503	0.00212	0.08642
CXCL13	6.8842	1.46E-05	0.007937	METRN	1.783864	0.0008	0.06058	PYGM	1.65863	0.0021	0.08642
C16orf89	6.5996	1.79E-05	0.009252	NR4A1	1.473594	0.0008	0.06058	SLC29A4	2.772068	0.00212	0.08642
HK3	3.3093	1.81E-05	0.009252	SLC45A4	1.473657	0.0008	0.06058	AKAP8L	1.244145	0.00214	0.08668
P2RX1	3.0256	1.90E-05	0.009417	UCP2	1.670025	0.0008	0.06058	BAIAP3	1.488168	0.00226	0.08795
CLIC6	3.9723	1.98E-05	0.009536	RASIP1	1.87647	0.0008	0.06077	CHD5	2.671492	0.00225	0.08795
LYPD6B	3.4246	2.03E-05	0.009536	PRR22	1.33516	0.0008	0.0609	CXCL12	1.41365	0.00225	0.08795
CD19	4.9209	2.13E-05	0.009737	BCRP3	2.867727	0.0008	0.06122	DPM2	1.44221	0.00223	0.08795
SHC2	3.2579	2.40E-05	0.010715	CYP3A4	-1.48872	0.0008	0.06122	HSD17B3	2.19505	0.00225	0.08795
AMH	1.5093	2.62E-05	0.011202	EMID1	3.125499	0.0008	0.06122	KIAA0513	0.973116	0.00219	0.08795
COL2A1	8.7257	2.65E-05	0.011202	FAM13A-A!	0.979115	0.0008	0.06122	LOC1002890	0.906796	0.00224	0.08795
KIAA0125	4.5555	2.71E-05	0.011202	DCAF6	-0.27093	0.0009	0.06145	MYEF2	1.926673	0.00224	0.08795
AZGP1	6.4459	3.13E-05	0.011848	KIRREL2	4.592594	0.0009	0.06145	NARFL	0.951734	0.00221	0.08795
C16orf79	2.1392	2.94E-05	0.011848	KRT20	2.971766	0.0009	0.06145	POMT2	0.489555	0.00226	0.08795
SIX2	6.0148	3.00E-05	0.011848	ZNF444	1.838047	0.0009	0.06145	PPARGC1A	1.458189	0.00223	0.08795
SPRR2B	-3.1879	3.13E-05	0.011848	PLIN4	1.906334	0.0009	0.0616	PTPRJ	1.94326	0.0022	0.08795
GKN1	4.268	3.29E-05	0.012186	CETN2	-0.53879	0.0009	0.06177	SAMD11	2.237832	0.00225	0.08795
KCNH2	3.694	3.49E-05	0.01264	LOC100506	4.232964	0.0009	0.06177	SLC1A2	3.495038	0.00223	0.08795
FABP3	6.8441	3.66E-05	0.012718	CMKLR1	1.448998	0.0009	0.0623	SLC43A2	1.610003	0.00222	0.08795
TFF2	5.413	3.60E-05	0.012718	ANO7	3.288883	0.0009	0.06337	SNPH	1.943313	0.00221	0.08795
C2CD4B	4.4228	3.87E-05	0.013164	C21orf2	1.818332	0.0009	0.06337	TMEM139	2.176288	0.00223	0.08795
C2CD4C	2.8962	4.01E-05	0.013164	CYP2D7P1	1.104376	0.0009	0.06338	TMEM17	-0.64539	0.00226	0.08795
PLIN5	3.2552	3.97E-05	0.013164	NR1H3	1.934187	0.0009	0.06348	ZNF692	0.979712	0.00227	0.08795
FER1L4	3.8427	4.18E-05	0.013207	EPPK1	1.996032	0.0009	0.06362	BCAR1	1.409547	0.00228	0.08806

IGLL5	5.0244	4.14E-05	0.013207	IDUA	1.789711	0.0009	0.06362	PILRB	1.098703	0.00228	0.08806
SPRR2F	-3.1807	4.31E-05	0.01338	PACSIN1	4.460772	0.0009	0.06408	RRBP1	1.379168	0.00228	0.08806
C5orf32	3.9403	4.58E-05	0.013448	ANO5	3.416966	0.0009	0.06422	COTL1	1.52326	0.00229	0.08821
CPA2	8.6255	4.42E-05	0.013448	CCL3	2.788352	0.001	0.06436	FBN2	1.624694	0.00232	0.08826
HEPACAM2	5.0369	4.61E-05	0.013448	HAGHL	1.875292	0.001	0.06436	LOC1006529	1.340553	0.00232	0.08826
HPN	6.2495	4.64E-05	0.013448	MGAT3	2.071906	0.001	0.06436	LOC338799	1.444399	0.00232	0.08826
COL4A3	3.0252	4.74E-05	0.013526	TBXA2R	2.248591	0.001	0.06436	NPAS3	3.471431	0.0023	0.08826
MGC16121	4.2533	5.35E-05	0.014821	KCNH6	4.663193	0.001	0.06487	PCDHGA5	-1.50014	0.00232	0.08826
MNX1	3.1522	5.37E-05	0.014821	PRICKLE2	0.947068	0.001	0.06487	ADAM23	-1.25894	0.00234	0.08849
AKR7A3	3.7981	6.21E-05	0.016108	BEX1	5.128104	0.001	0.06519	C19orf28	0.925526	0.00233	0.08849
CXCL1	3.8223	6.11E-05	0.016108	CADPS	3.029834	0.001	0.06519	CRELD1	1.153256	0.00234	0.08849
LCN12	2.8044	6.18E-05	0.016108	TMEM198	1.711631	0.001	0.06519	FEZ1	2.147846	0.00235	0.08849
TMED6	4.5605	6.19E-05	0.016108	CCDC78	1.983708	0.001	0.06562	LOC1005070	1.466694	0.00236	0.08852
LINC00092	4.9033	6.86E-05	0.017546	CDK10	1.304353	0.001	0.06562	SPATA20	1.789678	0.00235	0.08852
TMEM178	2.5858	7.01E-05	0.017663	FCRL2	3.477601	0.001	0.06562	SLC18A2	1.48559	0.00237	0.08872
GPT	2.6087	7.48E-05	0.018581	FGA	9.763845	0.001	0.06562	HIP1R	1.407013	0.00239	0.08955
APLP1	3.9758	7.87E-05	0.019275	LOC100506	1.295727	0.001	0.06562	TNNC2	1.737648	0.00242	0.09013
FCER2	5.87	8.39E-05	0.020275	MAP1A	2.298255	0.001	0.06562	ASPSCR1	1.425282	0.00245	0.09035
PKDCC	3.1786	8.61E-05	0.020508	MYL4	3.364447	0.001	0.06562	C9orf84	2.216549	0.00244	0.09035
ERN2	5.5212	9.40E-05	0.022093	PIGR	4.187357	0.001	0.06562	PIDD	1.247356	0.00243	0.09035
C1QTNF1	2.4631	9.55E-05	0.022154	ZGLP1	1.442766	0.001	0.06562	SIN3B	0.502756	0.00245	0.09035
ESRRG	4.5675	0.00011	0.024088	AATK	2.6259	0.001	0.06662	SLC25A34	1.104865	0.00244	0.09035
UBE2QL1	5.1553	0.00011	0.024088	PODXL2	1.819955	0.0011	0.06676	TBC1D30	1.944583	0.00245	0.09035
CDHR2	4.0278	0.00011	0.024735	ZDHHC11	1.846767	0.0011	0.06679	C9orf7	1.724838	0.00246	0.09037
VSIG1	3.9547	0.00012	0.02598	ANXA10	5.729819	0.0011	0.06705	LOC1002935	0.410875	0.00246	0.09037
APOC1	2.4848	0.00012	0.02638	CAMK2B	2.506718	0.0011	0.06705	SH2B2	1.188718	0.00247	0.09042
LOC283663	1.3315	0.00012	0.026664	DGKQ	0.709865	0.0011	0.06705	TESC	2.955355	0.00247	0.09042
EGR2	2.5854	0.00013	0.026763	FAM167B	2.221344	0.0011	0.06705	SIGIRR	1.618089	0.0025	0.09109
LRRC4B	2.6827	0.00013	0.026763	LGALS4	2.829913	0.0011	0.06705	FCRL3	3.163579	0.00251	0.09128
TNFRSF13B	4.565	0.00013	0.027228	SNX22	1.873753	0.0011	0.06705	SGSM3	1.166602	0.00252	0.09128
CAPN8	4.2423	0.00014	0.027737	TAZ	1.011287	0.0011	0.06705	TTC35	-0.55925	0.00252	0.09128
TNNC1	3.1531	0.00014	0.027737	C19orf45	3.152974	0.0011	0.06722	FZD8	1.850035	0.00254	0.09179
IGHMBP2	0.8886	0.00014	0.02798	KIF19	2.817264	0.0011	0.06722	SLC18A1	3.6516	0.00254	0.09179
ANPEP	2.1549	0.00015	0.028462	STAC3	0.861193	0.0011	0.06722	S1PR2	0.746717	0.00257	0.09236
C3orf45	4.8383	0.00015	0.028462	EXD3	1.84777	0.0011	0.06726	F2RL3	2.750496	0.00257	0.09243
DGCR8	0.6284	0.00015	0.028462	KCNJ12	2.087121	0.0011	0.06726	MST1P2	1.641576	0.0026	0.09308
KCNQ1	3.4513	0.00015	0.028462	LGALS1	1.981797	0.0011	0.06726	PNKP	0.78972	0.00261	0.09308
SERPINA1	2.514	0.00015	0.028462	PDE10A	1.226623	0.0011	0.06726	TREX2	-1.19181	0.00261	0.09308
SLC26A9	3.9874	0.00015	0.028462	RENBP	1.92685	0.0011	0.06726	ARSE	3.51246	0.00262	0.09311
TPSG1	3.7594	0.00015	0.028462	SGSM2	0.726181	0.0011	0.06726	GRASP	1.432192	0.00262	0.09311
HSH2D	2.6772	0.00016	0.029781	TPSAB1	2.084594	0.0011	0.06726	LOC1005055	2.858678	0.00263	0.09311
TMEM176A	2.902	0.00016	0.029781	TSPAN15	2.281941	0.0011	0.06726	PDGFD	2.33916	0.00262	0.09311
DAPK1	2.0977	0.00017	0.030155	WNK2	2.287088	0.0011	0.06726	PDIA5	1.872161	0.00264	0.09311
EXOC3L4	3.6706	0.00017	0.030155	LZTR1	0.618902	0.0012	0.06754	SIGLECP3	1.876451	0.00264	0.09311
GPC3	3.2625	0.00017	0.030155	SPPL2B	1.403486	0.0012	0.06754	SNRNP70	1.292043	0.00264	0.09311
LINGO4	3.2506	0.00017	0.030155	MDK	2.033052	0.0012	0.06836	CSPG4	2.149188	0.00268	0.09381
CLDN18	6.1619	0.00019	0.030246	PDGFA	1.859372	0.0012	0.06929	SLC38A10	0.958701	0.00267	0.09381
CPAMD8	2.9139	0.00019	0.030246	C19orf6	0.909622	0.0012	0.06941	C9orf128	3.766597	0.0027	0.09383
GRK5	2.0734	0.00018	0.030246	MSLNL	7.380827	0.0012	0.06941	CHPF	1.371865	0.0027	0.09383
LINC00261	7.1954	0.00019	0.030246	ZNF275	0.838743	0.0012	0.06941	EEF1B2	-0.63843	0.00269	0.09383
LOC1006529	1.6754	0.00019	0.030246	TNFRSF4	2.306314	0.0012	0.06943	NCF1C	1.262062	0.00269	0.09383
LOC389023	3.6534	0.00019	0.030246	DOT1L	0.612079	0.0012	0.07003	OSGIN1	1.204895	0.0027	0.09383
NME7	-0.7532	0.00019	0.030246	CRELD2	1.62878	0.0012	0.07016	AEBP1	1.8007	0.00272	0.09413
SH3GL2	6.4478	0.00019	0.030246	SEZ6L2	2.880822	0.0012	0.07016	RIMS1	3.548194	0.00272	0.09413
SYT5	5.7583	0.00018	0.030246	ITPKA	3.0107	0.0012	0.07019	AGPAT6	0.739192	0.00273	0.09419
TCAP	2.2711	0.00018	0.030246	SDSL	2.212146	0.0012	0.07019	DAPK2	1.660304	0.00274	0.09436
TFF1	4.6917	0.00018	0.030246	ADAP1	1.521789	0.0013	0.07047	HEATR8	2.639167	0.00276	0.09473
TMEM14A	-0.8804	0.00018	0.030246	MAFK	0.702678	0.0013	0.07047	CNTN4	1.91114	0.00278	0.09516
PP7080	2.251	0.0002	0.030753	ACCN3	1.525118	0.0014	0.07066	FAM110B	2.043741	0.00278	0.09516

GRAMD1C	-0.584	0.00021	0.031815	ACCS	1.490314	0.0013	0.07066	HES6	1.755917	0.00279	0.09516
TMEM86B	1.9975	0.00021	0.03187	ARC	-1.5467	0.0013	0.07066	C9orf142	1.268313	0.0028	0.09552
CCDC88B	0.9837	0.00022	0.032303	B3GAT1	3.829843	0.0013	0.07066	AGER	1.172423	0.00283	0.09576
KIAA1875	1.4007	0.00022	0.032303	CELF5	2.948032	0.0013	0.07066	C2CD4A	4.026775	0.00283	0.09576
NFE2	3.5683	0.00022	0.032303	COL23A1	2.178459	0.0014	0.07066	FBXL6	0.852694	0.00282	0.09576
GADD45G	2.4672	0.00023	0.033061	DACT3	2.240033	0.0014	0.07066	KRT18	1.341734	0.00283	0.09576
CCL19	3.262	0.00023	0.033082	LOC100129	1.644035	0.0013	0.07066	SIGLEC1	1.499406	0.00284	0.09576
LOC150776	0.8183	0.00023	0.033082	LOC100129	5.411786	0.0013	0.07066	ATF7IP2	1.458551	0.00284	0.0958
EMILIN2	1.9533	0.00023	0.033499	MATN4	2.260453	0.0013	0.07066	ARMCX3	0.882101	0.00288	0.09646
TNFRSF8	3.29	0.00024	0.034365	MUC5AC	4.786412	0.0014	0.07066	GUSBP11	1.26836	0.00287	0.09646
IGF2BP2	2.3051	0.00025	0.034917	MUC6	5.634321	0.0014	0.07066	LOC1001303	1.053731	0.00288	0.09646
PYY2	3.1586	0.00025	0.034917	NCAM1	3.317315	0.0014	0.07066	PIGZ	1.399883	0.00288	0.09646
INPP5J	2.0493	0.00026	0.035548	NPB	1.626799	0.0014	0.07066	ADAMTS10	1.28683	0.00292	0.09721
CHGA	5.9431	0.00026	0.036126	NRXN1	3.095047	0.0013	0.07066	CACNA1B	2.477612	0.00291	0.09721
GPBAR1	2.8941	0.00027	0.036517	ORAI2	0.7154	0.0013	0.07066	PALM	1.512596	0.00292	0.09721
LGALS9	2.1754	0.00027	0.036517	ORAOV1	0.509078	0.0013	0.07066	APOL1	1.764182	0.00303	0.09772
NEURL	3.2088	0.00028	0.037209	PARVB	1.81611	0.0013	0.07066	ATP6V0E2	1.357841	0.00304	0.09772
IGJ	3.892	0.00028	0.037316	PRTFDC1	2.271693	0.0013	0.07066	B4GALNT1	3.632862	0.00308	0.09772
GLYCTK	2.3137	0.00028	0.03736	RBPMS2	3.453428	0.0013	0.07066	C17orf56	0.984736	0.00302	0.09772
C17orf110	4.0475	0.00029	0.037432	RHOBTB3	2.227235	0.0013	0.07066	C1orf159	0.939457	0.00305	0.09772
ATP13A2	0.812	0.00031	0.038074	SIGLEC11	4.667576	0.0013	0.07066	CHST2	1.392245	0.00306	0.09772
BLK	3.6731	0.00031	0.038074	SLC29A1	1.382147	0.0013	0.07066	CKB	2.432488	0.00311	0.09772
C12orf28	3.1093	0.0003	0.038074	STAB1	1.283266	0.0013	0.07066	CLDN15	1.014773	0.00298	0.09772
C9orf150	2.4285	0.0003	0.038074	TFR2	1.976769	0.0013	0.07066	COL4A4	1.428031	0.003	0.09772
DIO3OS	4.8171	0.00031	0.038074	TIMP1	1.845087	0.0013	0.07066	FAM162A	-0.70286	0.00307	0.09772
FA2H	2.461	0.0003	0.038074	TP53I13	1.578903	0.0014	0.07066	FAM174B	1.658552	0.00311	0.09772
FAM20C	1.7963	0.00031	0.038074	TRIM3	1.34121	0.0014	0.07066	FAM65C	1.588859	0.00309	0.09772
FLJ44511	2.0594	0.00031	0.038074	VPREB3	2.725222	0.0014	0.07066	HSD17B6	1.99784	0.00301	0.09772
LTBP4	0.8382	0.00031	0.038074	GMPPA	1.515006	0.0014	0.07068	INCA1	1.474721	0.00299	0.09772
MC1R	1.8005	0.0003	0.038074	EFHD2	1.303256	0.0014	0.07119	LOC1001328	0.850334	0.00297	0.09772
REG1A	7.0422	0.00032	0.038074	ADCK1	1.422205	0.0014	0.07178	LOC1003026	1.12788	0.0031	0.09772
CPNE7	2.0461	0.00034	0.039179	LOC400643	-2.45502	0.0014	0.07179	LOC1006527	1.47735	0.00306	0.09772
DPP10	4.1159	0.00033	0.039179	LCN10	2.169334	0.0014	0.0719	MAPK8IP3	0.871428	0.00309	0.09772
RASSF7	1.7774	0.00033	0.039179	LOC100507	0.968838	0.0014	0.0719	NES	1.395267	0.00304	0.09772
TCIRG1	1.5978	0.00033	0.039179	LOC440993	1.445651	0.0014	0.0719	NPAS1	1.920799	0.00295	0.09772
TNFRSF17	4.5032	0.00034	0.039179	ProSAPiP1	1.836308	0.0014	0.0719	NTRK3	4.513865	0.00311	0.09772
ZNF579	2.1709	0.00034	0.039741	RBM14	0.398008	0.0014	0.0719	OSR1	2.960143	0.003	0.09772
PDE9A	2.1357	0.00035	0.040062	CNTD1	4.355193	0.0014	0.07195	PDLIM7	1.1897	0.00304	0.09772
C9orf152	2.1873	0.00036	0.040946	SPIRE2	2.040458	0.0014	0.07195	PIGC	-0.37164	0.0031	0.09772
LRRC24	2.0011	0.00037	0.042051	KIAA1530	0.876589	0.0015	0.07206	RARRES2	2.355814	0.00295	0.09772
SLC2A6	1.7543	0.00039	0.043341	C3orf32	2.459105	0.0015	0.0722	SCCPDH	1.483524	0.00305	0.09772
SYNPO	1.5251	0.00039	0.043341	LRRC20	-1.37219	0.0015	0.07297	SDK1	1.456729	0.00305	0.09772
MAMDC4	1.2766	0.00039	0.043465	CHRNA10	0.870279	0.0015	0.07343	SNORD104	1.429771	0.00297	0.09772
TMPRSS6	2.9397	0.00039	0.043465	DPP6	3.893732	0.0015	0.07345	SYN2	2.201154	0.00309	0.09772
HYAL1	2.1268	0.0004	0.044061	SDC2	2.339065	0.0015	0.07456	TMEM175	1.321816	0.00301	0.09772
ASRGL1	3.1237	0.00041	0.044762	ATAD3B	1.24331	0.0015	0.07483	ZDHHC8	0.477416	0.00309	0.09772
CBWD1	-0.7211	0.00041	0.044943	ITGA7	1.439586	0.0015	0.07501	CENPP	-0.65934	0.00314	0.09776
DHRS2	3.3364	0.00042	0.044943	MS4A7	1.338045	0.0015	0.07506	COL9A1	2.861499	0.00314	0.09776
CHIA	9.3383	0.00042	0.045451	C1orf186	2.127669	0.0016	0.07576	FAM160B2	0.622628	0.00315	0.09776
KCTD19	4.4493	0.00043	0.04549	C8orf82	1.02028	0.0016	0.07576	IRGQ	0.412355	0.00316	0.09776
LOC728743	2.2036	0.00043	0.04549	C8ORFK29	1.841445	0.0016	0.07576	LOC1005056	-2.09476	0.00314	0.09776
LOC100128(	1.9173	0.00044	0.046223	POFUT2	1.119347	0.0016	0.07576	MBL1P	2.482319	0.00316	0.09776
FAM109B	1.0332	0.00045	0.04667	RGS2	1.408287	0.0016	0.0767	NPFF	1.35005	0.00314	0.09776
SLC41A2	2.5794	0.00045	0.04667	TAGLN	2.787006	0.0016	0.07681	NPY1R	1.944365	0.00316	0.09776
CORO7	1.4823	0.00045	0.046859	LOC100507	1.08022	0.0016	0.07748	C10orf125	1.109003	0.0032	0.09785
RECQL4	1.7416	0.00046	0.046859	GLIS2	1.556839	0.0017	0.07781	C19orf21	2.631935	0.00321	0.09785
TNFRSF14	1.8232	0.00046	0.046859	LOC143188	0.904045	0.0016	0.07781	CCDC107	1.657773	0.00321	0.09785
MAPK11	2.041	0.00046	0.04695	MRVI1	1.684265	0.0016	0.07781	CCL18	3.958181	0.00323	0.09785
BRSK1	2.3585	0.00048	0.047442	ANKS3	1.309422	0.0017	0.07807	CLCN7	1.188827	0.00322	0.09785

CADPS2	1.8568	0.00047	0.047442	SOCS1	1.789454	0.0017	0.07833	EBI3	2.22993	0.00318	0.09785
INSRR	5.647	0.00049	0.047442	GAS2	2.503248	0.0017	0.07837	FASTK	0.821939	0.00325	0.09785
LHFPL2	1.9113	0.00048	0.047442	KCNK12	-2.78504	0.0017	0.07837	FCGRT	1.578904	0.00322	0.09785
LOC115110	2.5243	0.00047	0.047442	TSPAN8	3.494436	0.0017	0.07845	KCNJ11	2.474828	0.00326	0.09785
LOC145837	4.1221	0.00048	0.047442	ANO9	1.129055	0.0017	0.07851	KIAA1755	3.167604	0.00322	0.09785
STK33	3.3126	0.00048	0.047442	TMEM92	3.026092	0.0017	0.07851	MAPRE3	1.12304	0.00323	0.09785
QPCT	-0.9186	0.00051	0.049258	SLC44A4	3.120045	0.0017	0.07866	MTMR9LP	1.731022	0.00324	0.09785
TRPM4	1.2457	0.00051	0.049258	AMIGO3	1.001066	0.0017	0.07874	NBEA	2.200137	0.00325	0.09785
LYL1	1.8816	0.00052	0.049541	UST	-0.8107	0.0017	0.07924	PNKD	1.682733	0.00325	0.09785
SULT1C2	6.0395	0.00052	0.049691	LOC100652	1.165896	0.0017	0.07951	SAMD14	2.328584	0.00319	0.09785
CASP14	-2.2356	0.00053	0.04986	SLC4A2	1.519531	0.0017	0.07951	SDCCAG3	0.557595	0.00317	0.09785
CHTF18	0.7832	0.00053	0.04986	CYP39A1	-0.98052	0.0017	0.07961	XBP1	1.070275	0.0032	0.09785
TMEM125	1.9833	0.00053	0.04986	HEXDC	1.585991	0.0018	0.08003	CD244	-0.94611	0.00328	0.09817
YJEFN3	1.83	0.00053	0.04986	G0S2	2.52344	0.0018	0.08081	ORAI1	1.241005	0.00328	0.09817
BMPR1B	3.194	0.00054	0.050174	KCNMB1	2.039927	0.0018	0.08096	ACADS	1.50934	0.00331	0.0985
PDE6G	4.0209	0.00054	0.050174	PROS1	-0.9918	0.0018	0.08096	ARHGEF10	1.566689	0.0033	0.0985
C17orf107	1.8385	0.00055	0.050292	GPR133	2.319874	0.0018	0.0815	ELN	1.582333	0.0033	0.0985
FN3K	2.5029	0.00055	0.050292	LOC100129	2.424064	0.0018	0.0815	LUC7L	1.204195	0.00332	0.09857
IFT74	-0.6304	0.00056	0.050525	MFSD7	1.241346	0.0018	0.0815	FNDC5	4.282739	0.00333	0.09878
ZBTB46	1.039	0.00056	0.050525	KIF1A	5.106197	0.0018	0.08153	TMEM180	1.189161	0.00333	0.09878
FUT1	2.0089	0.00056	0.050532	FAM193B	1.038607	0.0018	0.08172	ARHGDIG	2.633022	0.00335	0.09889
ADAM33	2.6197	0.00057	0.050672	LOC100505	1.201501	0.0018	0.08172	ARRB2	1.085175	0.00336	0.09889
ASPHD1	3.4746	0.00057	0.050672	LOC100652	1.585144	0.0019	0.08219	GNAZ	2.820278	0.00336	0.09889
CREB3L1	3.6421	0.00057	0.050672	NWD1	2.190875	0.0019	0.08234	GOLGA3	0.536307	0.00335	0.09889
ADCY5	1.466	0.00058	0.051229	RN7SL1	1.983745	0.0019	0.08234	NCKAP5L	0.788586	0.0034	0.09986
CAPS	2.0051	0.00059	0.051229								
